# Rapid Expansion of Phenylthiocarbamide Non-Tasters among Japanese Macaques

**DOI:** 10.1371/journal.pone.0132016

**Published:** 2015-07-22

**Authors:** Nami Suzuki-Hashido, Takashi Hayakawa, Atsushi Matsui, Yasuhiro Go, Yoshiro Ishimaru, Takumi Misaka, Keiko Abe, Hirohisa Hirai, Yoko Satta, Hiroo Imai

**Affiliations:** 1 Molecular Biology Section, Department of Cellular and Molecular Biology, Primate Research Institute, Kyoto University, Inuyama, Aichi, Japan; 2 Japan Society for the Promotion of Science, Tokyo, Japan; 3 Graduate School of Agricultural and Life Sciences, The University of Tokyo, Tokyo, Japan; 4 Department of Evolutionary Studies of Biosystems, The Graduate University for Advanced Studies (Sokendai), Hayama, Kanagawa, Japan; Barnard College, Columbia University, UNITED STATES

## Abstract

Bitter taste receptors (TAS2R proteins) allow mammals to detect and avoid ingestion of toxins in food. Thus, TAS2Rs play an important role in food choice and are subject to complex natural selection pressures. In our previous study, we examined nucleotide variation in *TAS2R38*, a gene expressing bitter taste receptor for phenylthiocarbamide (PTC), in 333 Japanese macaques (*Macaca fuscata*) from 9 local populations in Japan. We identified a PTC “non-taster” *TAS2R38* allele in Japanese macaques that was caused by a loss of the start codon. This PTC non-taster allele was only found in a limited local population (the Kii area), at a frequency of 29%. In this study, we confirmed that this allele was present in only the Kii population by analyzing an additional 264 individuals from eight new populations. Using cellular and behavioral experiments, we found that this allele lost its receptor function for perceiving PTC. The nucleotide sequences of the allele including flanking regions (of about 10 kb) from 23 chromosomes were identical, suggesting that a non-taster allele arose and expanded in the Kii population during the last 13,000 years. Genetic analyses of non-coding regions in Kii individuals and neighboring populations indicated that the high allele frequency in the Kii population could not be explained by demographic history, suggesting that positive selection resulted in a rapid increase in PTC non-tasters in the Kii population. The loss-of-function that occurred at the *TAS2R38* locus presumably provided a fitness advantage to Japanese macaques in the Kii population. Because TAS2R38 ligands are often found in plants, this functional change in fitness is perhaps related to feeding habit specificity. These findings should provide valuable insights for elucidating adaptive evolutionary changes with respect to various environments in wild mammals.

## Introduction

**Fig 1:**
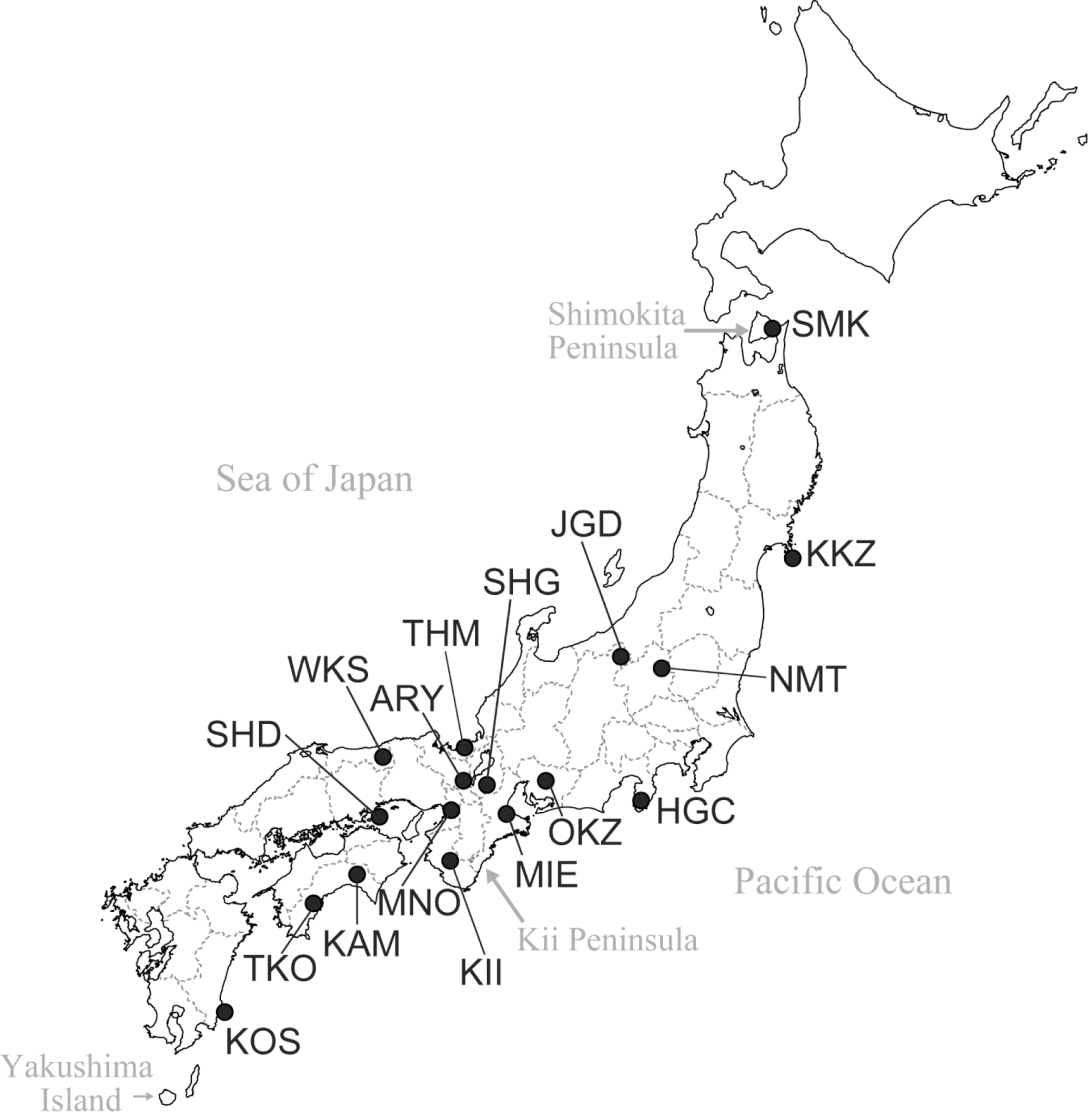
Sampling sites for Japanese macaques. The 17 sampling sites are indicated by black circles with their abbreviated locality name (Table 1).

Japanese macaques (*Macaca fuscata*) are widely distributed from the northern part of the main island of Japan (Shimokita Peninsula) to the southern island of Japan (Yakushima Island) (Fig 1), and thus occupy the northernmost habitats of all non-human primates. Japanese macaques adapted to the Japanese environment after they diverged from rhesus macaques (*M*. *mulatta*) approximately 0.31–0.88 million years ago (mya) [1] and can survive even in the snowy environments of mountainous areas or in northern areas where the temperatures reach below −20°C during the winter. Therefore, they have suitable food habits for survival in variable environments [2].

The genetic variability within and among various local populations of Japanese macaques has been well studied [3–5]. A phylogenetic study of mitochondrial DNA (mtDNA) showed that Japanese macaques that live in eastern Japan have lower genetic diversity than those living in western Japan. This was attributed to an ancient population expansion that occurred in the eastern habitats after the last glacial period approximately 15,000 years ago [6]. A population genetic study of blood protein polymorphisms showed that genetic variation in Japanese macaques is not uniformly distributed and that variants are shared only in limited areas [4]. The characteristics are maintained by frequent exchanges of reproductive males between local populations. A previous study showed that different selection pressures have acted on the gene encoding Toll-like receptor 2, which plays an important role in the recognition of a variety of pathogenic microbes, between Japanese macaques and rhesus macaques, reflecting adaptation of the species to different habitats [7]. Thus, other functional genes, particularly those involved in perceiving environmental signals or stimuli, may also vary among habitats within Japanese macaques, assuming that they are adapted to particular environments in Japan. However, such variation in functional genes has not been fully characterized with respect to environmental variation.

The sense of taste allows mammals to evaluate consumed food. Among the five recognized taste sensations (sweet, bitter, sour, salty, and umami), bitter taste plays an important role in avoiding ingestion of toxins. In mammals, bitter taste is mediated by a family of seven-transmembrane G protein-coupled receptor genes, *TAS2Rs* [8,9]. Humans and mice have 26 and approximately 40 functional *TAS2R* genes, respectively, and some loci exhibit intraspecific variation including nonfunctional alleles [10–14]. Because *TAS2Rs* are directly involved in food choice, the number of *TAS2R* genes in the genome has changed frequently during mammalian evolution [13–17]. Some studies have suggested that TAS2R function varies, even within species, owing to different selection pressures depending on the habitat [18,19].

TAS2R38 is the best-studied bitter taste receptor in terms of intraspecific phenotypic variation [20,21]. It is a receptor for the synthetic bitter compounds phenylthiocarbamide (PTC) and propylthiouracil (PROP). Among natural bitter compounds, TAS2R38 recognizes glucosinolates and limonin, which are found in cruciferous and citrus plants, respectively [22]. Individual variation in human ability to taste PTC was first recognized in 1931 [23]. Such phenotypic variation has subsequently been found in many other primates, including chimpanzees (*Pan troglodytes*) [24,25]. Most phenotypic variation among humans is caused by three amino acid substitutions in TAS2R38, whereas for chimpanzees the observed variation results from a start codon mutation (ATG>AGG) in *TAS2R38* [20,21]. Premature stop codons have been identified in brown wooly monkeys (*Lagothrix lagotricha*) and black-handed spider monkeys (*Ateles geoffroyi*) [26]. These mutations are predicted to disrupt receptor function. Although it is not clear whether these disruptive mutations in the wooly monkey and spider monkey are polymorphic or fixed, these findings indicate that multiple primate species have phenotypic variation in PTC sensitivity.

Furthermore, it has been suggested that different types of natural selection have acted on *TAS2R38* in humans and chimpanzees. In humans, balancing selection may have maintained phenotypic variation resulting from “taster” and “non-taster” alleles at the *TAS2R38* locus [27]. In contrast, in chimpanzees, the non-taster allele was found in only western chimpanzees (*P*. *troglodytes verus*) where it was present in 76% of individuals, while it was not found in eastern chimpanzees (*P*. *troglodytes schweinfurthii*) [19]. In addition, an *F*_ST_ analysis suggested that different types of selective pressures have acted on western chimpanzees and eastern chimpanzees. Therefore, the evolutionary mechanisms underlying the phenotypic variation associated with TAS2R38 in multiple primate species may be related to their diets and habitats.

In our previous study, we determined *TAS2R38* sequences for 333 Japanese macaques and 55 rhesus macaques. We identified a PTC “non-taster” allele in Japanese macaques that was caused by a start codon mutation (ATG>ACG) using nucleotide sequence comparisons and qualitative behavioral tests [28]. Interestingly, the non-taster allele was restricted to the Kii population and had a frequency of 29% in this population. However, the evolutionary history of this non-taster allele remains uncertain. In the present study, we confirmed that individuals with the PTC non-taster phenotype were only in the Kii population using many more samples, and quantitatively evaluated the causal mutation leading to a nonfunctional TAS2R38 receptor. Furthermore, we obtained evidence of positive selection on the non-taster *TAS2R38* allele in the Kii population. This positive selection in a restricted region of Japan may ultimately reveal that adaptive evolution in Japanese macaques depended on their habitat.

## Results

### *TAS2R38* genetic variation among Japanese macaques

We previously identified a non-taster allele, designated *Mf-K*, with a start codon mutation (ATG>ACG) that occurred in only the Kii population [28]. In this study, to investigate whether other local populations also had the *Mf-K* allele, we analyzed many more samples and determined sequence variation in *TAS2R38* (1002 bp, including 239 synonymous sites and 760 nonsynonymous sites). We analyzed a total of 597 Japanese macaques including 264 new and 333 previously identified individuals from 17 local populations (Table 1). Single nucleotide variants (SNVs) found among these 597 Japanese macaques included 3 synonymous and 12 nonsynonymous substitutions, for a total of 15 variable sites.

**Table 1:** *TAS2R38* variants in Japanese macaques and rhesus macaques.

	Population Name	Abbreviation	Population Type^a^	*n* ^b^	*S* ^c^	*h* ^d^	*π* ^e^	*θ* ^f^	*D* ^g^
Japanese macaques	Shimokita	SMK	(3)	83	3	4	0.083	0.053	0.94
	Kinkazan	KKZ	(3)	9	0	1	0.000	0.000	NA
	Numata	NMT	(3)	20	3	4	0.038	0.070	-1.01
	Takahama^h^	THM	(1)	27	5	6	0.168	0.110	1.26
	Jigokudani	JGD	(2)	40	4	4	0.072	0.081	-0.22
	Hagachi^h^	HGC	(2)	15	3	3	0.065	0.076	-0.34
	Okazaki	OKZ	(3)	4	2	3	0.100	0.077	1.10
	Mie	MIE	(3)	78	9	10	0.174	0.160	0.21
	Shiga^h^	SHG	(3)	37	6	8	0.167	0.123	0.86
	Arashiyama^h^	ARY	(1)	29	4	3	0.065	0.086	-0.54
	Minoo^h^	MNO	(2)	41	7	5	0.088	0.140	-0.90
	Kii^h^	KII	(2)	40	8	6	0.118	0.161	-0.67
	Wakasa^h^	WKA	(1)	41	8	7	0.224	0.160	0.99
	Shodoshima^h^	SHD	(1)	11	1	2	0.017	0.027	-0.64
	Kami	KAM	(3)	12	6	8	0.247	0.160	1.64
	Takaoka	TKO	(3)	29	3	2	0.072	0.065	0.24
	Koshima^h^	KOS	(2)	81	4	4	0.113	0.071	1.09
	All			597	15	20	0.142	0.195	-0.60
rhesus macaques	China^h^	CHN	(1)	27	7	7	0.265	0.153	1.89
	India^h^	IND	(1)	27	5	5	0.156	0.110	1.02
	All			54	8	10	0.236	0.152	1.33

^a^Population types: (1) captive-born populations that retained their locality attribution, (2) wild-born populations, and (3) wild populations.

^b^Number of individuals.

^c^Number of polymorphic sites.

^d^Number of haplotypes.

^e^Nucleotide diversity per site (%).

^f^Watterson’s *θ* per site (%).

^g^Tajima’s *D*. Two-sided Tajima’s *D* test was performed using coalescent simulations with 10,000 replicates, assuming no recombination and a Poisson distribution of mutations along lineages. All Tajima’s *D* values were not significant (*P* ≥ 0.05).

^h^Reported in our previous study [28].

**Fig 2:**
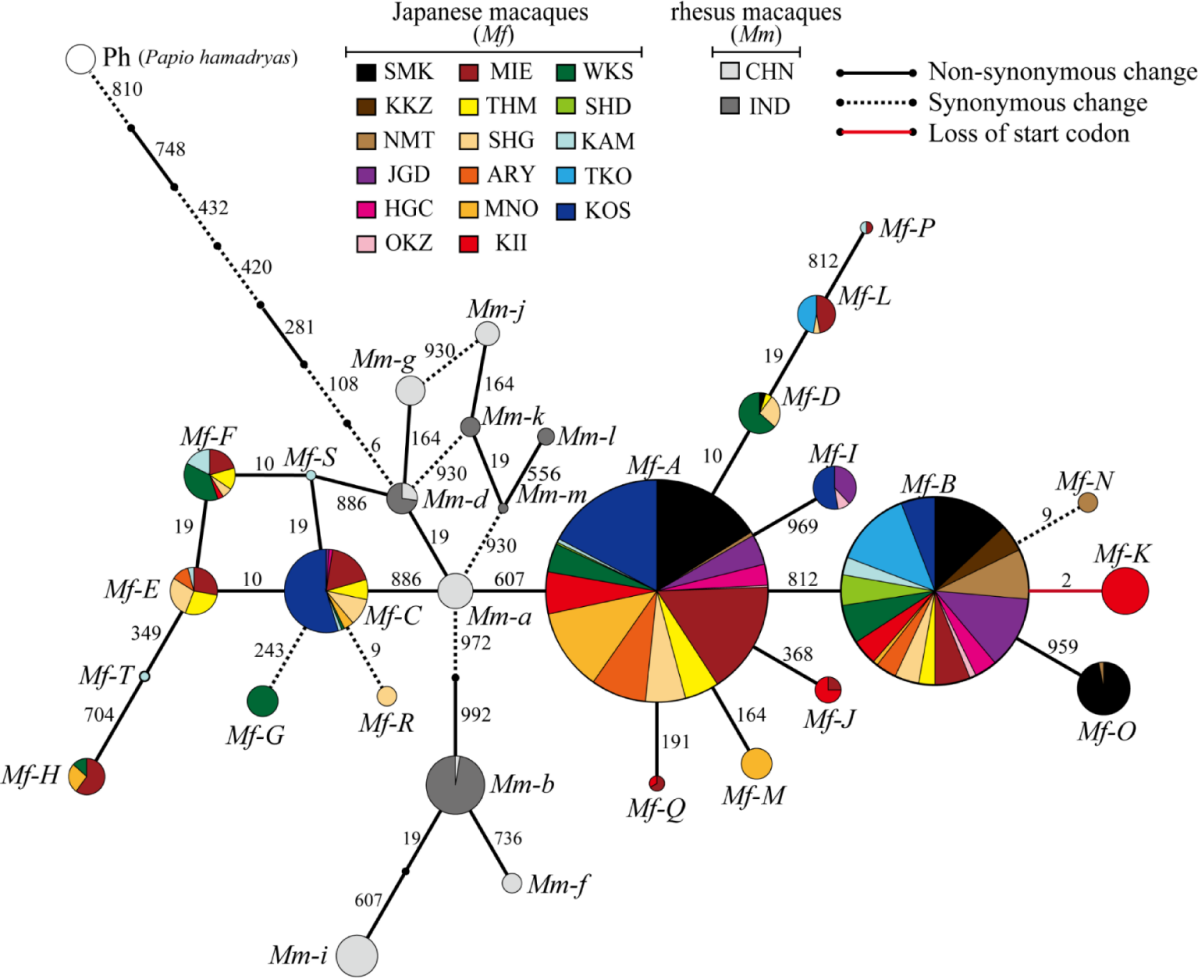
Median-joining network for *TAS2R38* alleles of Japanese and rhesus macaques. Each circle represents a different haplotype and is shown along with the allele name. *Mf* and *Mm* indicate Japanese and rhesus macaque haplotypes, respectively. Colors within a circle indicate each local population (Table 1), and the size of a circle is proportional to the number of chromosomes. Nucleotide positions of mutations that differentiate alleles are indicated on the network branches. Line styles of branches indicate mutation types.

We identified 20 alleles based on combinations of these 15 SNVs and designated them *Mf-A–T* (S1 Table). This included 7 new alleles that were not identified in our previous study. These 20 alleles were categorized into three groups (Fig 2, S2 Table). The first group included only the *Mf-A* and *Mf-B* haplotypes; it comprised many populations that were widely distributed and the haplotypes retained a high frequency in each local population. The second group included the *Mf-G*, *Mf-K*, *Mf-M*, *Mf-N*, *Mf-R*, *Mf-S*, and *Mf-T* alleles. These alleles were identified as population-specific. Eleven residual alleles were included in the third group; they were shared by several populations with a low frequency in each local population.

Because Japanese macaques diverged from rhesus macaques 0.31–0.88 mya [1], their nucleotide sequences are very similar and any phylogenic relationships among alleles from both species are expected to be intermingled. To elucidate the phylogenetic relationships among these *TAS2R38* alleles, we constructed a network of 20 *TAS2R38* alleles, including 10 rhesus macaque alleles identified in our previous study (Fig 2). This network showed that the Japanese and rhesus macaque alleles were not completely differentiated; rather, the Japanese macaque sequences formed 2 clusters that diverged from *Mm-a*, a rhesus macaque allele. However, the non-taster *Mf-K* allele clustered separately from rhesus macaque alleles and arose independently in Japanese macaques after the species divergence.

Although we investigated 8 additional local populations in this study, we did not detect the *Mf-K* allele in any of the other local populations. Thus, we confirmed that *Mf-K* occurred in only the Kii population. Interestingly, the frequency of the *Mf-K* allele was 29% in the Kii population, whereas the other alleles of the second group (*Mf-G*, *Mf-M*, *Mf-N*, *Mf-R*, *Mf-S*, and *Mf-T*) had frequencies of <13% in each of the local populations (S2 Table).

### Functional analysis of TAS2R38 variants using cultured cells

**Fig 3:**
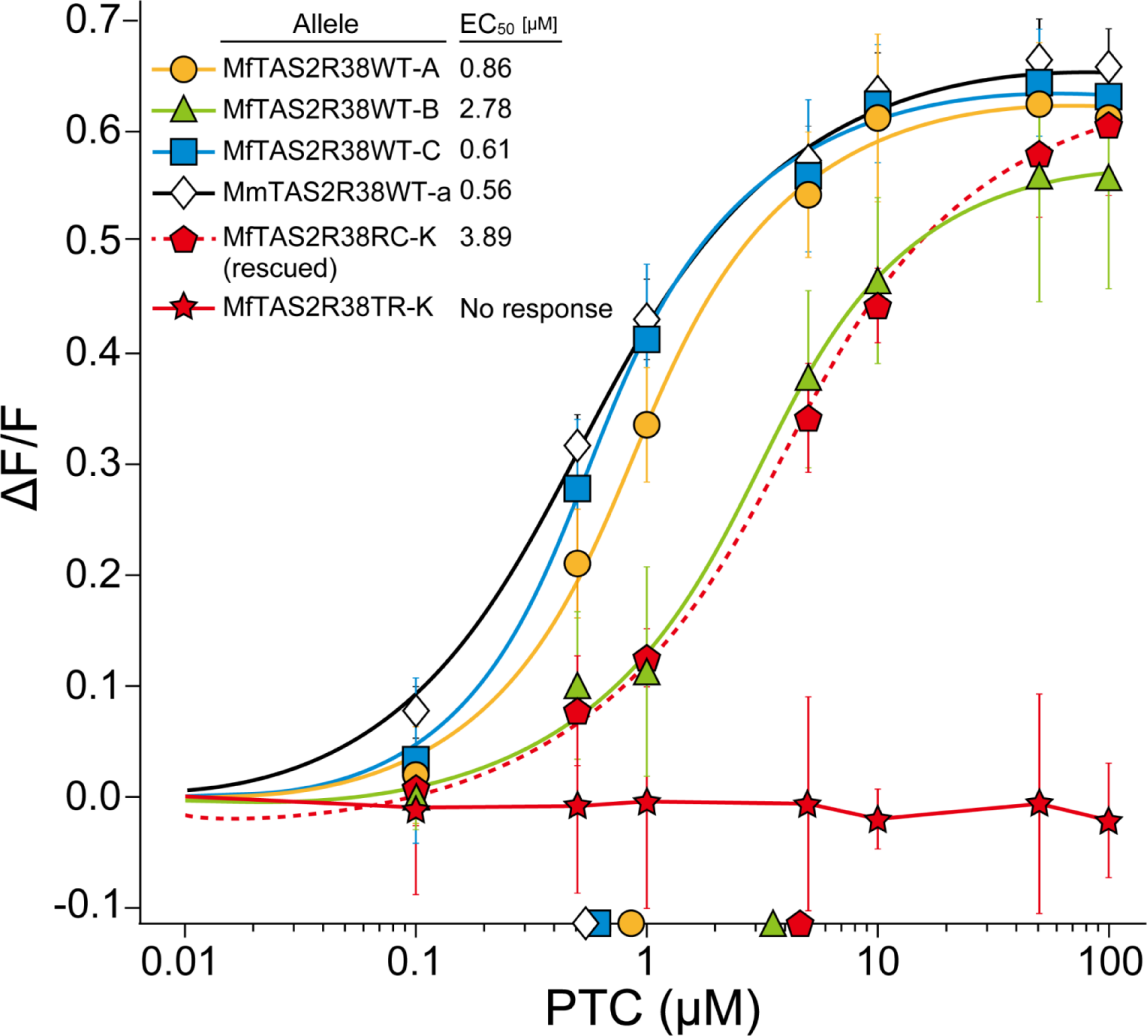
Dose-response curves for intracellular Ca^2+^ levels in cells that express TAS2R38 variants against PTC concentrations. Each point represents the mean ± standard error determined from 3 independent measurements. Points on the horizontal axis indicate the EC_50_ values for alleles.

In our previous study, we showed that *Mf-K* homozygous individuals had a lower sensitivity to PTC than those individuals lacking the *Mf-K* allele. In this study, we quantitatively assessed the receptor activity of the high-frequency alleles, including *Mf-K*. We performed functional assays using PTC as the ligand and a calcium imaging method. We first tested whether a mutation in the start codon altered the receptor activity. Four receptor variants—MfTAS2R38WT-A,-B,-C, and MmTAS2R38WT-a, all of which were predicted to be full-length receptors—responded to PTC (Fig 3). In contrast, MfTAS2R38TR-K, which lacked 96 amino acids in the TAS2R38WT receptor owing to a missense mutation in the start codon, failed to respond to PTC. An MfTAS2R38RC-K variant that was engineered to have a start codon upstream of the native start site detected PTC, suggesting a complete rescue of receptor function. These results showed that Mf*TAS2R38-A*,*-B*,-*C*, and *MmTAS2R38-a* produced a functional receptor, but the Mf*TAS2R38-K* allele did not.

To determine the effects of amino acid variation on receptor sensitivity, we compared the responsiveness of receptor variants, MfTAS2R38WT-A,-B,-C, and MmTAS2R38WT-a, by calculating their EC_50_ values (half maximal effective concentration values; see Materials and methods). The EC_50_ value of MfTAS2R38WT-B was 3.2–5.0 times higher than those of MfTAS2R38WT-A,-C, and MmTAS2R38WT-a (Fig 3). There were no differences among the latter three variants. A modified MfTAS2R38RC-K that was engineered to have an upstream start codon had an EC_50_ value comparable to that of MfTAS2R38WT-B. Because both MfTAS2R38WT-B and MfTAS2R38RC-K had a common amino acid substitution at position 271 from isoleucine to threonine (S1 Fig), this amino acid substitution was responsible for the slight decrease in TAS2R38 receptor sensitivity. An amino acid substitution at position 203, which was the only difference between MfTAS2R38WT-A and-C, did not influence sensitivity to PTC.

### Behavioral experiments

**Fig 4:**
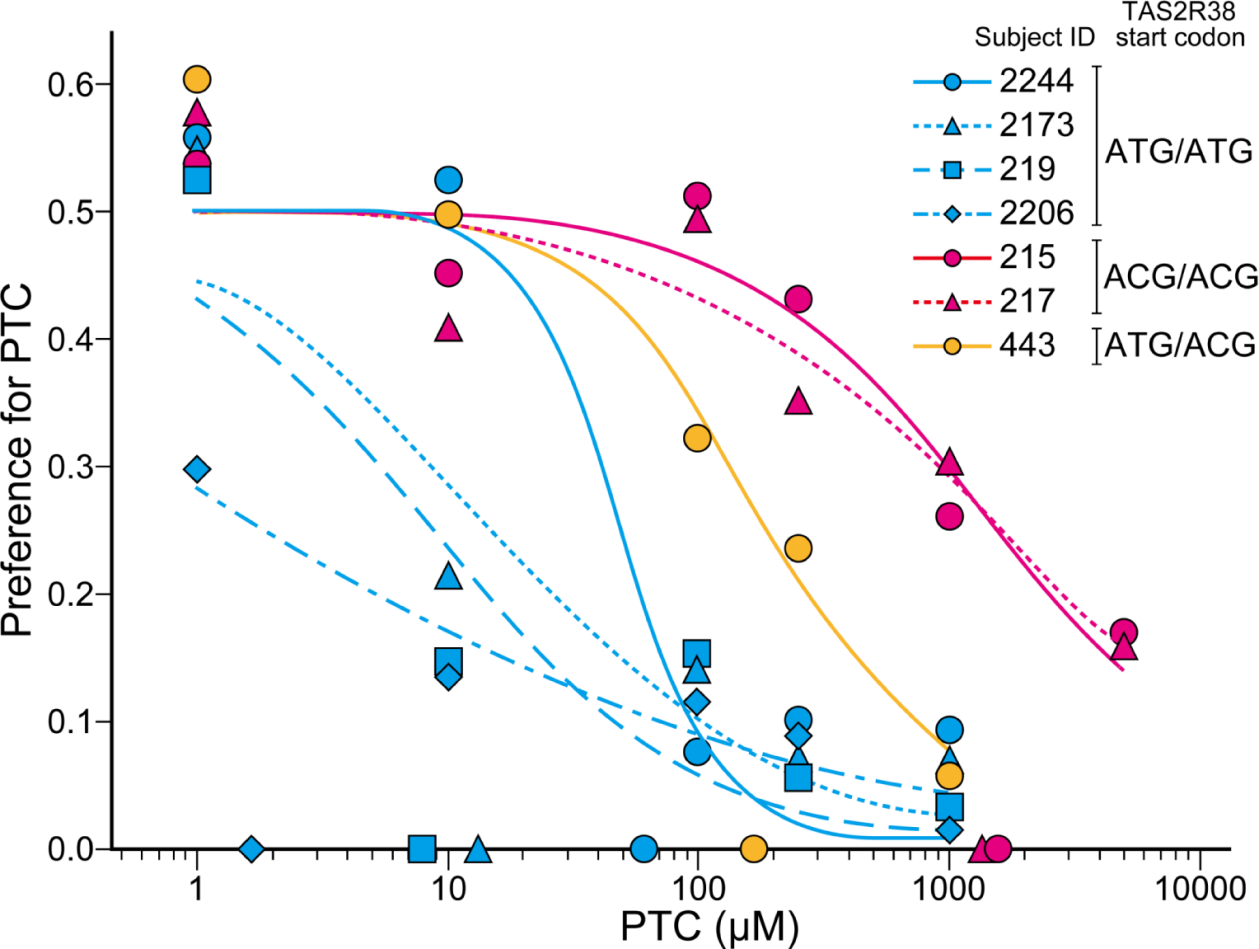
Gustatory responsiveness of Japanese macaques to various PTC solution concentrations. The vertical axis indicates the preference ratio for PTC, which is the ratio of PTC solution consumed to the total liquid consumed. Each point represents the mean of 6 trials. Point and line colors indicate the genotype of the start codon for *TAS2R38*. Points on the horizontal axis indicate the EC_50_ values for individuals. The responsiveness of each individual is shown in the (S2 Fig).

In our previous study, we found qualitative differences in sensitivity between 4 and 3 individuals with ATG/ATG and ACG/ACG homozygous *TAS2R38* start codons, respectively [28]. We used a two-bottle preference test with PTC solutions of various concentrations to quantify the differences in sensitivity thresholds between these 2 homozygotes (Fig 4, S2 Fig). We calculated the EC_50_ values that indicate the PTC concentration at which the preference ratio becomes 25%, half of the level due to chance, and compared these values between the 2 homozygotes. The average EC_50_ value for ATG/ATG individuals was 18.9 μM with a large standard error (±22.1). This result was consistent with results obtained for humans, for which PTC thresholds showed considerable variability within the “taster” group [29]. In contrast, the average EC_50_ value for ACG/ACG individuals was 1542.1 μM (±8.9), which was significantly higher than that of ATG/ATG individuals (80-fold).

We also used this test for an individual that was an ATG/ACG heterozygote at the *TAS2R38* start codon and obtained an EC_50_ value of 179.2 μM (Fig 4). This was intermediate between the values of each homozygote and was consistent with human studies showing that taster/non-taster heterozygote individuals (PAV/AVI genotype) had intermediate sensitivity between taster and non-taster groups [30].

### Analysis of *TAS2R38* flanking regions

In our genetic analysis of *TAS2R38*, population-specific alleles, except for the *Mf-K* allele, had frequencies of <1% overall in Japanese macaques and frequencies of <13% in each local population (S1 and S2 Tables). It is expected that population-specific alleles would be maintained at a low frequency in each population because they probably arose after population differentiation. However, the *Mf-K* allele had a frequency of 29% in the Kii population and its distribution was distinctly different from that of the other alleles. To clarify the evolutionary history of *TAS2R38* alleles found in the Kii population, we sequenced *TAS2R38* flanking regions and determined non-coding SNVs linked to each *TAS2R38* allele.

We determined the nucleotide sequences for the *TAS2R38* flanking region (10,231 bp) in all 40 individuals sampled from the Kii population. The *TAS2R38* allele distributions for these sequenced samples were 32 *Mf-A*, 17 *Mf-B*, 1 *Mf-F*, 6 *Mf-J*, 23 *Mf-K*, and 1 *Mf-Q*. The overall nucleotide diversity (*π*) values for the 5′ (4229 bp), coding (1,002 bp), and 3′ (5000 bp) regions were 0.49%, 0.12%, and 0.06%, respectively. These alleles comprised 13 long-range haplotypes and were named for the *TAS2R38* coding region with distinguishing numbers, such as *A-1*, *A-2*, *B-1*, and *B-2* (S3 Fig). There were 7 and 2 different haplotypes for *Mf-A* (*A-1*–*A-7*) and *Mf-B* (*B-1* and *B-2*) alleles, respectively. All *Mf-K* haplotype sequences for the 23 chromosomes were identical. For other alleles, only 1 haplotype was detected for each allele *(F-1*, *J-1*, and *Q-1*).

We compared the informative sites of these 13 haplotypes and investigated their phylogenetic relationships. There were numerous incompatible sites in the 5′- and 3′-flanking regions (S3 Fig). This strongly suggested that genetic recombination occurred at a site located between 2 polymorphic sites: positions 179,408,639 and 179,408,483 of chromosome 3. Indeed, the topology and branch lengths of the tree for the 5′ region, including position 179,408,639, were different from those of the 3′ region, including position 179,408,483. Therefore, we excluded a possible 5′-recombinant region when we conducted a haplotype network analysis. Then, we determined the phylogenetic relationships based on *TAS2R38* using the 6411 bp sequence, including the *TAS2R38* coding region (S3 Fig).

**Fig 5:**
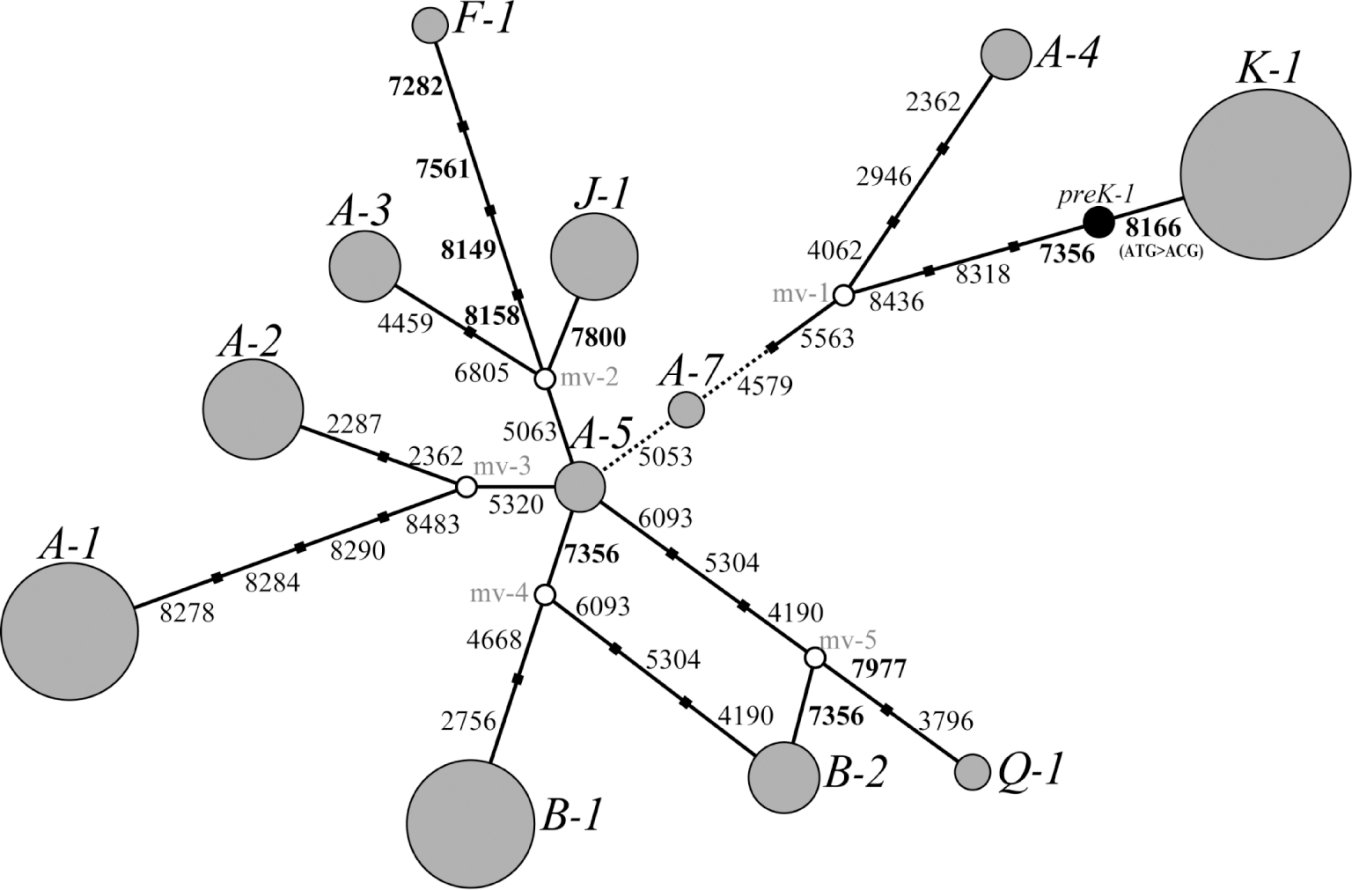
Median-joining network for *TAS2R38* flanking region haplotypes in the Kii population. Gray and white circles represent the flanking haplotypes and median-vectors, respectively. The sizes of gray circles are proportional to the numbers of chromosomes. The numbers along branches indicate nucleotide positions of mutations that distinguish haplotypes and correspond to the last 4 digits of the positions in the rheMac2 genome sequences (S3 Fig). Bold numbers indicate mutations in the *TAS2R38* coding region and the others indicate mutations in non-coding regions. Dashed lines indicate indel mutations. A substitution numbered 8166 indicates a mutation at the start codon.

We constructed a haplotype network for *TAS2R38* flanking regions to determine when and how the *Mf-K* allele arose (Fig 5). The presumable most recent common ancestral haplotype of *K-1* and *A-4* designated median-vector-1 (mv-1) was derived from the *A-5* haplotype by obtaining 2 insertion-deletion (indel) mutations and 1 substitution (Fig 5). *K-1* then uniquely acquired 4 substitutions, including a nonsense mutation at the start codon. Of the 4 unique substitution sites, 2 were located in the 5′ region and the others were located in the coding region. We analyzed whether these 4 substitutions occurred in 6 other neighboring local populations and identified the most similar haplotype, designated “*preK-1*”, in the JGD and HGC populations. The haplotype sequence was identical to that of the *K-1* haplotype, except for a mutation at the start codon (Fig 5). However, the *preK-1* haplotype was not found in the sampled Kii population.

The *K-1* haplotype was presumably derived from *preK-1* by acquiring a mutation at the start codon in the limited 6411-bp region that excluded regions of recombination. It is expected to take 160,000 years to acquire a single mutation in this 6411-bp sequence assuming a mutation rate of 10^−9^/site/year. This may be an overestimation because our data were limited to these 6411-bp. If we subsequently find no differences between *K-1* and *preK-1* in a longer sequence, this would indicate an earlier estimated divergence time between these 2 haplotypes. Furthermore, the *K-1* haplotype expanded to 23 chromosomes without acquiring any mutations in this 10-kbp sequence. When the *K-1* haplotype expanded to 23 chromosomes for *t* years under a star phylogeny, the total branch length was 23*t* years. For a mutation rate of 10^−9^/site/year, the mutation rate *λ* for a 10-kbp region for 23*t* years is 23*t* × 10^−5^. The assumption of a star phylogeny is conservative for this time estimation. Based on our data, no mutations occurred for 23*t* years. If the probability of this was set to >5%, then based on a Poisson distribution for the number of substitutions in this region, the upper limit for the time would be approximately 13,000 years (*t* < 13,024).

### General genetic characteristics of the Kii population

Japanese macaques generally have high migration rates between neighboring troops, and the breeding structure of this species follows the two-dimensional stepping-stone model developed by Kimura and Weiss [31]; genetic diversity within groups has been maintained by gene flow [4]. However, the *Mf-K* allele was not found in other local populations despite the frequency of *Mf-K* being 29% in the Kii population. This may be explained by the genetic specificity (i.e., a small effective population size or a low migration rate between neighboring populations) of the Kii population or natural selection for the *Mf-K* allele of *TAS2R38*. Therefore, we analyzed the sequences of other non-coding regions in the Kii and 7 neighboring populations to evaluate their genetic diversity and migration level.

We determined the sequences of 9 loci in non-coding regions (average length, 725 bp/locus) in the Kii and 7 additional neighboring populations and compared these sequences within each population (Table 2 and S3 Table). The average nucleotide diversity (*π*) over 9 loci in the Kii population was 0.076% and was similar to that of the other 7 populations, 0.087% (SD: 0.017%). Additionally, to investigate the demography of the Kii population, we estimated Tajima’s *D*, which provides an index of an effect of population dynamics including demography and selection (S3 Table). Although most of these values were not significantly different from zero, the *D* value at the *IGS09* locus was significantly positive in the Kii population. However, this tendency was also found in the other populations, except for the HGC population. This showed that this tendency, observed in all 8 populations including the Kii population, was inherited from an ancestral population.

**Table 2:** Average genetic diversity of 9 loci in non-coding regions.

Population Name	*S* ^a^	*π* ^b^	*θ* ^c^
Takahama	1.2	0.059 (0.09)	0.050 (0.07)
Jigokudani	2.3	0.105 (0.09)	0.100 (0.08)
Hagachi	2.9	0.101 (0.10)	0.119 (0.10)
Mie	2.0	0.082 (0.13)	0.082 (0.10)
Shiga	2.7	0.101 (0.12)	0.112 (0.10)
Arashiyama	1.6	0.073 (0.09)	0.066 (0.06)
Minoo	1.8	0.088 (0.11)	0.074 (0.09)
Kii	1.7	0.076 (0.10)	0.069 (0.07)
Average	2.0	0.085	0.084

^a^Average number of polymorphic sites per locus.

^b^Average nucleotide diversity per site (%), with standard deviation in parentheses.

^c^Average number of segregating sites per site (%), with standard deviation in parentheses.

**Fig 6:**
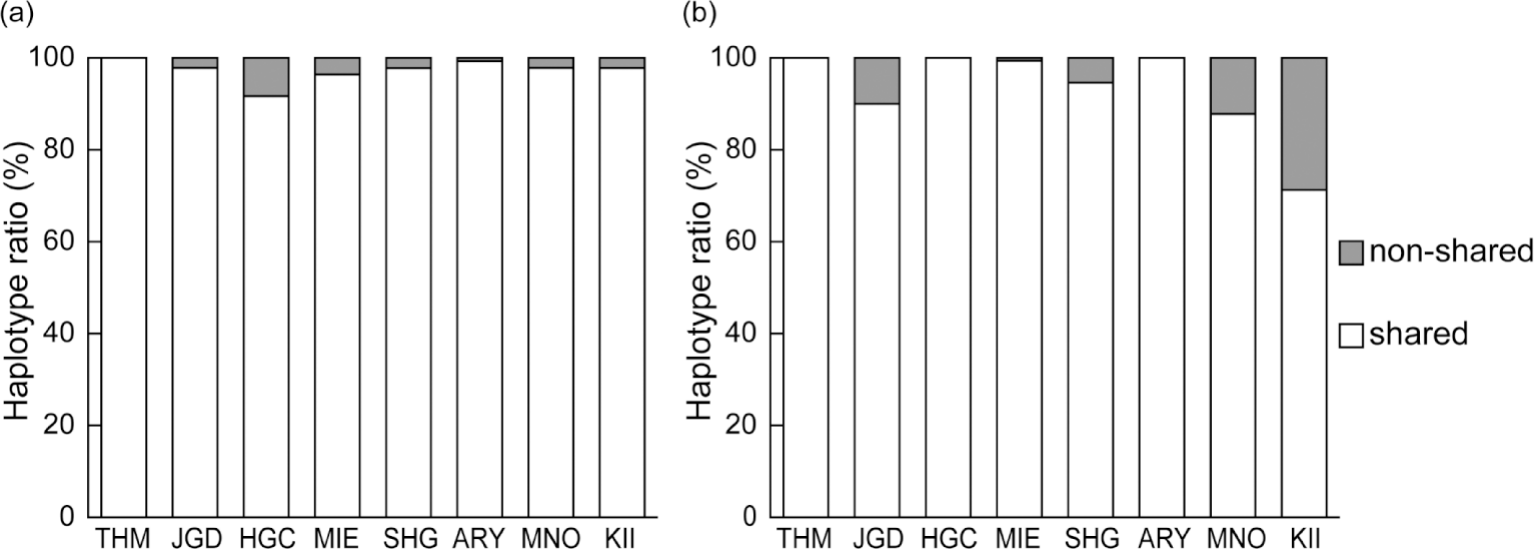
Proportion of shared and non-shared haplotypes among 8 Japanese macaque populations. Proportions of shared and non-shared haplotypes (a) in 9 loci in non-coding regions and (b) in *TAS2R38* coding regions. Gray and white bars indicate the rates of non-shared and shared haplotypes, respectively.

To evaluate the effects of gene flow between these local populations, we calculated the *F*_ST_ value for each of the 8 populations in a pairwise manner (Table 3). The average differentiation of 9 loci in the Kii population was 0.08 and was similar to the average of the other 7 populations, 0.11. We also calculated the *Nm* values, where *N* is the effective population size and *m* is the migration rate. If *Nm* > 1, then local differentiation is less pronounced. In particular, if *Nm* ≥ 4, then the entire population tends to behave as a single panmictic population, assuming the two-dimensional stepping-stone model [32]. The *Nm* value for the Kii population was 5.6, which suggested high migration between the Kii and neighboring populations. We also separately calculated the proportions of shared haplotypes at 9 non-coding regions and the *TAS2R38* coding region (Fig 6). In non-coding regions, the average proportion of specific haplotypes in the Kii population was 5.15%, and 94.85% of haplotypes were shared with other populations. This indicated that the Kii population was not genetically isolated from these other populations or that this population was not sufficiently differentiated from the other populations.

**Table 3:** Pairwise migration levels for Kii and 7 neighboring populations.

		*Nm* ^b^								
		THM	JGD	HGC	MIE	SHG	ARY	MNO	KII	average
*F*_ST_ ^a^	Takahama		1.50	0.98	2.41	4.34	1.38	1.86	3.27	2.25
	Jigokudani	0.14		3.88	2.46	1.95	0.90	1.38	2.19	2.04
	Hagachi	0.20	0.06		2.00	1.65	1.05	1.14	1.75	1.78
	Mie	0.09	0.09	0.11		6.06	1.78	1.53	12.02	4.04
	Shiga	0.05	0.11	0.13	0.04		3.57	4.17	15.49	5.32
	Arashiyama	0.15	0.22	0.19	0.12	0.07		2.63	2.20	1.93
	Minoo	0.12	0.15	0.18	0.14	0.06	0.09		2.09	2.11
	Kii	0.07	0.10	0.12	0.02	0.02	0.10	0.11		5.57
	average	0.12	0.13	0.14	0.09	0.07	0.13	0.12	0.08	

^a^Pairwise *F*_ST_ values.

^b^Pairwise *Nm* values were calculated as follows: *F*_ST_ = 1/(1 + 4*Nm*).

In contrast, the proportion of shared haplotypes in the *TAS2R38* coding region was 71%, and that of region-specific haplotypes was 29%, which corresponded to the *Mf-K* frequency. Although such population-specific *TAS2R38* haplotypes were found in other populations, these proportions were relatively low (0.6%–12%). It would be very rare for a population-specific haplotype to have expanded to almost one-third of a particular population, and this characteristic appeared to be unique to the *TAS2R38* locus in the Kii population.

### Computer simulations for allele expansion

**Fig 7:**
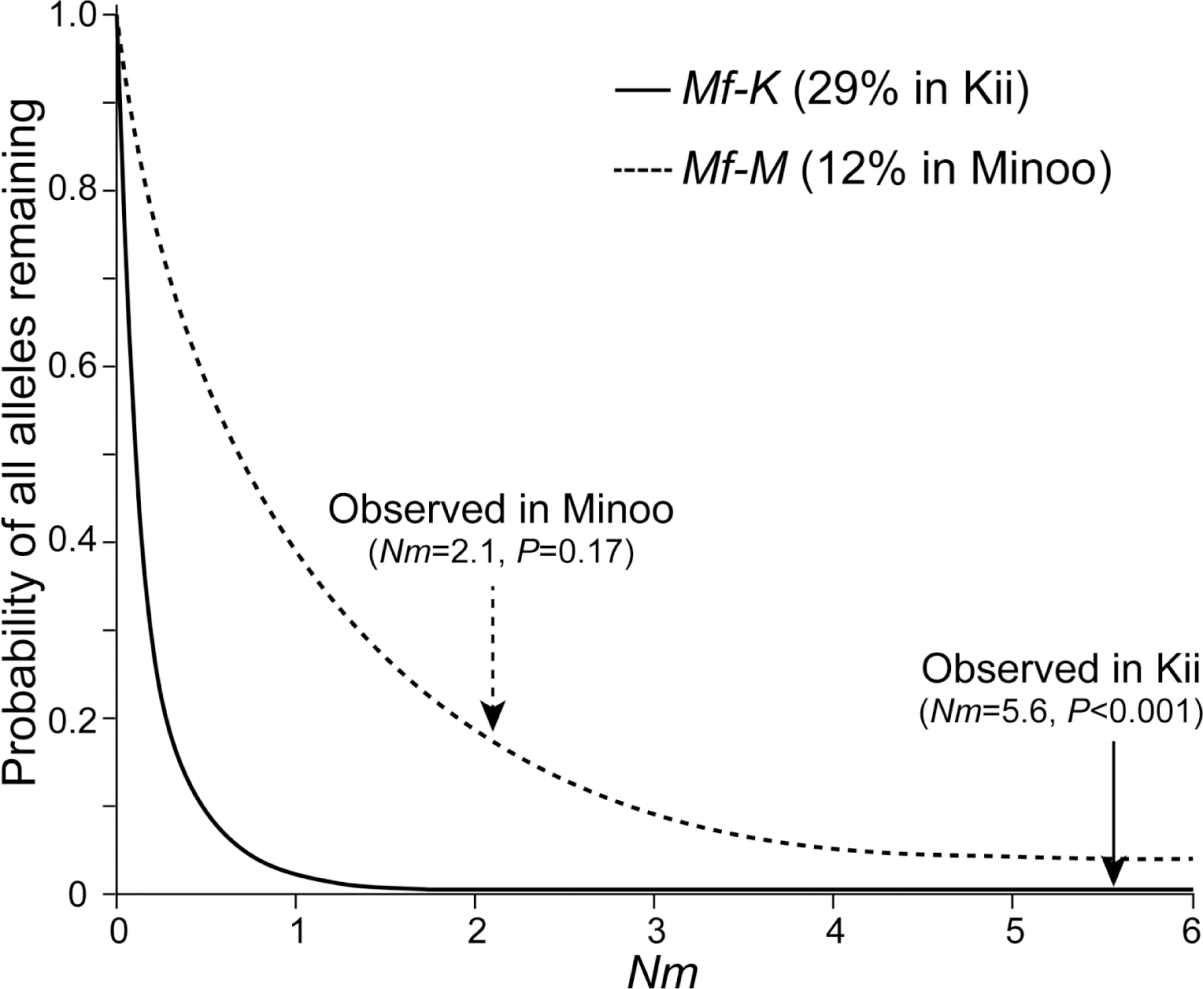
Computer simulations for migration among populations. The horizontal axis indicates the *Nm* value used as an index of the migration level. Graphs indicate the probability that all alleles remain in each population during expansion to each frequency with 1000 replications. A value of 0 indicated that all alleles migrated to other populations. Straight and dashed lines indicate *Mf-K* alleles in the Kii population and *Mf-M* alleles in the Minoo population, respectively, with each *Nm* value estimated from a non-coding region (Table 3) indicated by arrows.

The *Mf-K* allele, a non-taster allele, was found in only the Kii population at a frequency of 29%. Because this allele was associated with the loss of receptor function owing to a missense mutation at the start codon, the *Mf-K* allele could have expanded neutrally in the Kii population. We examined whether a neutral allele could increase in frequency to 29% without migration by computer simulations using various *Nm* values (Fig 7). The number of replicates used in these simulations was 1000. The estimated time for a neutral allele to increase in frequency to 29% without migration was 0.69*N* generations, where *N* is the effective population size. We confirmed that this value was consistent with the expected value using the formula in Kimura and Ohta [33].

The nucleotide sequence analyses of 9 neutral regions showed that the average *Nm* value was 5.6 (SD: 5.7) with a range of 1.8 < *Nm* < 15.5 between the Kii and neighboring populations (Table 3). These simulation results showed that a lack of migrants of a particular allele during 0.69*N* generations for all migration rates of >1.8 was unlikely (*P* < 0.005). This suggested that the absence of *Mf-K* alleles in the neighboring populations could not be explained by neutral processes (Fig 7). We also conducted these simulations for the Minoo population, which had a population-specific allele, *Mf-M*, at a frequency of 12%; this was the highest frequency for a population-specific allele, except for *Mf-K*, among our samples. The expected time for a neutral allele to expand to 12% was 0.27*N* generations, and the average *Nm* value between the Minoo and other populations was 2.1 (SD: 1.0). Based on these values, the occurrence of *Mf-M* at 12% without any migration under neutrality was not rejected (Fig 7).

Thus, only the frequency of the *Mf-K* allele could not be explained by neutral processes, which suggested that the *Mf-K* allele had expanded rapidly in a much shorter time than expected under neutrality. The time required for expanding to 29% in a neutral case, 0.69*N* generations, is 84,000 years when applying an effective population size and a generation time of Japanese macaques of 20,000 [34] and 6 years, respectively. The lack of mutations among the 23 *Mf-K* alleles showed that it expanded in the Kii population for 13,000 years at most, much shorter than 84,000 years. The most reasonable explanation for these data is that positive selection on the non-taster Mf-K phenotype promoted rapid expansion of the *Mf-K* allele.

## Discussion

In the present study, we found that positive selection operated on a loss-of-function *TAS2R38* mutation in Japanese macaques. Loss-of-function mutations that provide a selective advantage have been reported in humans, such as the delta-32 (Δ32) mutation in the chemokine receptor 5 (CCR5) gene. Because CCR5 is related to human immunodeficiency virus (HIV) infection and this 32-bp deletion results in a non-functional protein, individuals with this deletion have a selective advantage with respect to HIV resistance. It was estimated that this deletion arose and expanded within the past few thousand years [35].

A loss-of-function that has a selective advantage rather than a deleterious effect is curious, and it is of interest to determine the timing of this mutation in *TAS2R38* and to evaluate the evolutionary history. Our results suggested that the non-taster allele *Mf-K* arose and expanded in the Kii population approximately 13,000 years ago. Mutations that caused phenotypic variation for tasting PTC have been identified in other primate species as well as in Japanese macaques [20,21]. A non-taster *TAS2R38* allele in humans arose 0.3–1.6 mya and has been maintained by balancing selection [27,36]. For chimpanzees, a non-taster allele was found only in western chimpanzees, and this allele has expanded after the diversification between eastern and western chimpanzees approximately 0.5 mya [19].

The times for the emergence of non-taster alleles differed among these 3 species, including the Holocene epoch for Japanese macaques, the Pleistocene epoch for humans, and after the Pleistocene epoch for chimpanzees. Therefore, ecological factors related to the expansion of the non-taster allele by positive selection in Japanese macaques may also be different from those of the other 2 species. For Japanese macaques, the specific characteristics of the feeding habits and habitat of the Kii population may have affected this expansion. The evolutionary and ecological factors that are related to non-taster *TAS2R38* variation in other primate species should be examined carefully by focusing on species-specific properties, as well as common factors and processes of expansion beyond species.

TAS2R38 enables the recognition of glucosinolates in cruciferous plants and limonin in citrus plants as natural bitter compounds [22], and Japanese macaques often eat some parts of these plants [2]. A previous study on humans showed that people who are homozygous for a sensitive allele (PAV/PAV) have a higher sensitivity to the bitterness of glucosinolate-producing vegetables than those who are homozygous for insensitive alleles (AVI/AVI), as well as their sensitivity to PTC. In addition, people who are heterozygous for these alleles have intermediate sensitivity [37]. In the present study, we revealed that Japanese macaques are divided into 3 types with regard to PTC sensitivity based on TAS2R38 genotypes, as in humans. Therefore, Japanese macaques could have a similar tendency as humans with respect to sensitivity to bitterness in cruciferous plants. Although the contribution of different *TAS2R38* genotypes to sensitivity to limonin bitterness remains unclear, it is also possible that TAS2R38 genotypes in Japanese macaques affect the citrus feeding behavior. The first citrus species in Japan was *Citrus tachibana* (After its Japanese common name, “*tachibana*”), which has grown wild for 2800 years. It is thought that the original site of *C*. *tachibana* growth was the Kii peninsula, which includes the habitat of the Kii population of Japanese macaques analyzed in the present study [38,39]. Agriculture over the past several hundred years has rapidly expanded the distribution of most cruciferous (e.g., cabbage and radish) and citrus plants, which are ingested by wild Japanese macaques [2]. Such expansion of these plants may be related to the rapid expansion of the non-taster allele among Japanese macaques.

Other geological factors should also be taken into consideration. The Kii peninsula has been struck by tsunamis at 400–600 year intervals [40]. One study reported that there was no significant change in the number of long-tailed macaques (*M*. *fascicularis*) after the earthquake in the Indian Ocean off Sumatra in 2004, where coastal forests were washed away by the ensuing tsunami [41]. Therefore, a decrease and transition in available food with dramatically changing vegetation due to a tsunami in the Kii area may have also favored individuals with low sensitivity to a particular bitterness.

Monkeys in the Kii population with the non-taster allele are housed in open enclosures at the Primate Research Institute, Kyoto University; accordingly, in future studies we can observe changes in behavior and fitness in response to various candidate plants (e.g., cruciferous plants and citrus fruits). In preliminary experiments, we provided citrus fruits including mandarin oranges and *C*. *tachibana* to Japanese macaques in individual cages. Monkeys often peeled, removed the seeds, and ate relatively large fruits, but did not peel or eat whole fruits when we provided small fruits (2–4 cm) such as *C*. *tachibana*. These results suggest that non-taster individuals have an advantage in terms of eating small fruits, presumably because they contain higher concentrations of limonin, which is a ligand for TAS2R38, in their peels and seeds. Although we need to account for changes in vegetation other factors, these preliminary results provide insight into the fitness advantage of PTC non-tasters in the Kii population of Japanese macaques.

A previous study that examined blood protein polymorphism in Japanese macaques showed that major alleles are distributed throughout Japan and that specific mutation types are confined to limited areas [4]. The *TAS2R38* allele distributions investigated in the present study were mostly consistent with the results of this previous study. However, a study by Nozawa [4] indicated that some mutants are not restricted to a certain local population, but are shared among some adjacent regions owing to migration. This pattern was not consistent with the distribution of the non-taster *Mf-K TAS2R38* allele, which was found only in the Kii population, despite its high frequency in this population.

The Kii population is located in the western Kii peninsula. Thus, we also investigated *TAS2R38* polymorphisms in the Mie population, which is located in the eastern Kii peninsula, to compare the genetic variation among these adjacent populations (Fig 1). The pairwise *F*_ST_ value for these 2 populations in the non-coding region was only 0.02 (SD: 0.045), which suggested that Japanese macaques in this area migrate frequently between these 2 populations. This was also supported by *TAS2R38* allele distributions in these 2 neighboring populations; 5 of 6 alleles in the Kii population were shared with the Mie population. However, the *Mf-K* allele was not found in the Mie population despite its high frequency in the Kii population, which suggested that the *Mf-K* allele had expanded more rapidly in the Kii population than it had spread to the Mie population by migration. Although the population structure of *TAS2R38* was generally similar to those of blood proteins in a previous study, only the non-taster allele *Mf-K* exhibited specific characteristics in terms of its distribution and frequency in the Kii population.

In *TAS2R38* flanking regions, the nucleotide diversity (*π*) of the excluded region within the 5′ region (3,820 bp) was 0.50% and this was approximately 10 times higher than that of the non-coding region (0.076%) and the 3′-flanking region (0.055%) of *TAS2R38*. Additionally, Tajima’s *D* for this region was significantly positive, 2.36 (*P* < 0.01), which indicated that there were an excess of haplotypes with intermediate frequencies. Furthermore, the divergence time (2.5 mya) between these haplotypes was much longer than the time since speciation between Japanese and rhesus macaques, 0.5 my. Shared ancestral polymorphisms may have been maintained among these macaque species rather than within Japanese macaques. Perhaps this 5′ region affects TAS2R38 expression by providing diversity to the *TAS2R38* control region. Accordingly, we only focused on the start codon mutation that occurred in a restricted manner in the Kii population. Other factors likely to affect TAS2R38 receptor function are mentioned above. Thus, we must investigate other possibilities.

We can postulate that wild mammals adapted to various environments by altered molecular mechanisms as well as learning. This is supported by our results showing that a loss-of-function mutation for specific bitter tastes was acquired by Japanese macaques in a specific population with region-specific vegetation and that this mutation expanded rapidly under positive selection.

## Materials and Methods

### Ethics statement

This study was carried out in strict accordance with recommendations in the Guidelines for Care and Use of Nonhuman Primates Version 2 and 3 of the Primate Research Institute, Kyoto University (2002, 2010). This guideline was prepared based on the provisions of the Guidelines for Proper Conduct of Animal Experiments (June 1, 2006; Science Council of Japan), Basic Policies for the Conduct of Animal Experiments in Research Institutions under the Jurisdiction of the Ministry of Health, Labor and Welfare (effective on June 1, 2006; Ministry of Health, Labor and Welfare (MHLW)), Fundamental Guidelines for Proper Conduct of Animal Experiment and Related Activities in Academic Research Institutions (Notice No. 71 of the Ministry of Education, Culture, Sports, Science and Technology (MEXT) dated June 1, 2006), and Standards Relating to the Care and Management of Laboratory Animals and Relief of Pain (Notice No. 88 of the Ministry of the Environment dated April 28, 2006). The experiments were approved by the Animal Welfare and Animal Care Committee (Monkey Committee) of the Primate Research Institute (2010-066, 2011-093, 2012-047).

### Samples

A total of 597 Japanese macaques from 17 local populations in Japan were studied, including 333 samples analyzed for the *TAS2R38* locus in our previous study [28] and 264 additional individuals from 8 populations. These 17 local populations were divided into 3 categories: (1) captive-born populations that retained their locality attribution, (2) wild-born populations, and (3) wild populations (Table 1). Because both (1) and (2) populations were kept in the Primate Research Institute, Kyoto University, blood samples were obtained when these macaques underwent health status check-ups. Monkeys were housed in open enclosures at the Primate Research Institute, Kyoto University, and lived with their mothers and group members. Monkeys received water ad libitum and received leaves, seeds, fruits, and insects in addition to pellets. Tissue, blood, or DNA samples of individuals belonging to populations in category (3) were provided during the course of government population management or population censuses. No animals were sacrificed for the purposes of the present study; however, monkeys in some populations were culled previously as a pest-control measure against crop damage, with the permission of the local government. Genetic and morphological data from these monkeys are utilized for population census used by various institutes (universities, museums, non-profits, etc.). Tissue sample from this group were used with the cooperation of the respective organizations. For monkeys in the government census, body measurement, blood collection, and application of a GPS telemetry device, and release were performed by a veterinarian belonging to a non-profit organization aimed at Japanese macaque conservation and management. Blood samples were used with the cooperation of this organization.

### *TAS2R38* sequencing and haplotype inferences

Genomic DNA was extracted from blood or tissue samples using the Quick Gene DNA Whole Blood Kit S (Fujifilm, Tokyo, Japan) or the DNeasy Blood & Tissue Kit (Qiagen, Hilden, Germany). To amplify and sequence the entire coding region of the Japanese macaque *TAS2R38* gene, primers were designed based on the whole-genome sequences of rhesus macaques (MGSC Merged 1.0/rheMac2) from the University of California, Santa Cruz website (http://genome.ucsc.edu/) using software available on the Primer3Plus website (http://www.bioinformatics.nl/primer3plus/). The primer sequences and the PCR conditions used are shown in S4 Table. PCR mixtures (25 μl total) contained 0.625 U of ExTaq DNA polymerase (Takara Bio Inc., Shiga, Japan), 2 mM reaction buffer and 0.2 mM deoxynucleoside triphosphates provided by the DNA polymerase manufacturer (Takara), primers (0.2 μM each), and an adequate amount of genomic DNA as the template. Amplification was performed using the following conditions: initial denaturation at 94°C for 10 min; 35–40 cycles of denaturation at 94°C for 10 sec, annealing at 56°C for 30 sec, and extension at 72°C for 1 min, followed by a final extension at 72°C for 10 min. PCR products were checked by electrophoresis in 1% agarose gels and purified by isopropanol precipitation and/or ExoSAP-IT (Affymetrix Inc., Santa Clara, CA, USA).

Using the PCR primers and internal primers, the purified PCR products were directly sequenced in both strand orientations with a BigDye Terminator v3.1 Cycle Sequencing Kit and a 3130 Genetic Analyzer (Applied Biosystems, Foster City, CA, USA). Chromatograms were imported to the ATGC software (Genetyx Corporation, Tokyo, Japan) and analyzed. Haplotype sequences were reconstructed from diploid sequence sets using PHASE v2.1 [42,43]. During reconstruction, sequences with sites inferred to have probabilities of <0.95 were excluded. Sequences of these unphased haplotypes were determined by cloning using a TOPO TA Cloning Kit (Invitrogen Corporation, Carlsbad, CA, USA). The *TAS2R38* coding sequences determined in the present study were deposited in GenBank under the accession numbers AB907224–AB907230.

### *TAS2R38* population and sequence analyses

Common population parameters were estimated using DnaSP v5.1 [44]. To summarize diversity levels, pairwise nucleotide diversity (*π*) and Watterson's *θ* based on the number of segregating sites were calculated [45,46]. Tajima’s *D* was also calculated to assess *TAS2R38* neutrality [47]. Median-joining networks of evolutionary relationships among the alleles were constructed, rooted with the *TAS2R38* sequence of the hamadryas baboon (*Papio hamadryas*; accession number AY724835.1) using NETWORK v4.6 [48].

### Functional analysis using cultured cells

To investigate the PTC responsiveness of each TAS2R38 variant, a functional assay with the major alleles found in Japanese and rhesus macaques, *Mf-A*, *Mf-B*, *Mf-C*, *Mf-K*, and *Mm-a*, was used with a calcium imaging method [49]. The PCR products for these alleles were tagged at the last 8 amino acids of bovine rhodopsin (bRh) at the C-terminal end. Subsequently, the tagged *TAS2R38* fragments and the first 45 amino acids of rat somatostatin receptor type 3 (ssr3) were ligated into a pEAK10 expression vector (Edge Biosystems, Gaithersburg, MD, USA) using Geneart (Invitrogen) to enhance cell-surface expression (S1 Fig).

Chimpanzees also have a start codon mutation (ATG>AGG) in *TAS2R38*, and the receptor activity of this allele was tested using a truncated polypeptide resulting from translation initiation at a downstream ATG codon (M97) described by Wooding et al. [21]. Thus, Mf*TAS2R38-K* that produced MfTAS2R38TR-K (truncated type) with 96 amino-terminal residues missing compared with wild-type MfTAS2R38WT was constructed (S1 Fig). As a control, Mf*TAS2R38*-*K* (rescued) that produced MfTAS2R38RC-K (rescued type) resulting from translation initiation at an upstream ATG codon in ssr3 was constructed (S1 Fig). All plasmids were sequenced to verify mutations and to exclude plasmids with amplification errors during PCR. Expression plasmids were used for the chimeric G-protein subunit G_α_16gust44, which was subcloned into pcDNA 3.1 (Invitrogen), to be co-expressed with TAS2R38 [50].

Human embryonic kidney 293T (HEK293T) cells were cultured at 37°C in Dulbecco’s modified Eagle’s medium (DMEM; Sigma-Aldrich, Tokyo, Japan) supplemented with 10% fetal bovine serum (Life Technologies, Grand Island, NY). HEK293T cells were kindly provided by Dr. Hiroaki Matsunami (Department of Neurobiology and Duke Institute for Brain Sciences, Duke University Medical Center). For transfection, cells were seeded in 35-mm dishes and transiently transfected with plasmids that expressed each recombinant TAS2R38 along with G_α_16gust44 using Lipofectamine 2000 (Invitrogen). Transfected cells were transferred to a 96-well lumox multiwell plate (SARSTEDT AG & Co., Nümbrecht, Germany) 6 h after transfection and then incubated for an additional 18–20 h. Wells were rinsed with an assay buffer (130 mM NaCl, 10 mM glucose, 5 mM KCl, 2 mM CaCl_2_, 1.2 mM MgCl_2_, and 10 mM HEPES; pH 7.4) and loaded with 5 μM fluo-4 AM (Dojindo Laboratories, Kumamoto, Japan). Plates were then incubated for 45 min at 27°C in the dark. Subsequently, a plate was placed on a FlexStation 3 (Molecular Devices, Inc., Sunnyvale, CA, USA) for fluorescence detection. Changes in fluorescence intensity were monitored at 2-s intervals. After baseline readings for 20 s, PTC (Sigma-Aldrich) dissolved in assay buffer was added and scanning resumed for an additional 100 s.

Fluorescence signals were evaluated as *F* values. A response was expressed as the Δ*F*/*F* value [Δ*F*/*F* = (*F* − *F*_*0*_)/*F*_*0*_], which was the normalized peak response relative to background fluorescence (*F*_*0*_). Based on the mean Δ*F*/*F* value at each concentration across 3 independent experiments, these data were fit to the equation: *f(x)* = *I*_min_ + [*I*_max_ − *I*_min_/(1 + (*x*/EC_50_)^*h*^], where *x*, EC_50_, and *h* represent the ligand concentration, the half maximal effective concentration value, and the Hill coefficient, respectively. Curve fitting and parameter estimations were done using the non-linear least squares method (Levenberg–Marquardt algorithm) implemented in R v2.14.0 (http://www.r-project.org/).

### Two-bottle preference test

Behavioral tests were performed for 7 Japanese macaques from the Kii population, which were housed in individual cages at the Primate Research Institute, Kyoto University. Of these 7 macaques, 4 had the ATG/ATG genotype, 1 had the ATG/ACG genotype, and 2 had the ACG/ACG genotype at the start codon of the *TAS2R38* gene. Monkeys were kept in an air-conditioned room in individual cages (90 cm wide, 76 cm length, 85 cm height). For environmental enrichment, monkeys were able to play with toys (wood blocks, chains, and cords) and interacted with other monkeys visually and vocally. All monkeys were given fruits and sweet potatoes in addition to pellets. Monkeys received water ad libitum during the two-bottle preference test; the automatic water supply was disabled and distilled water and PTC solutions were instead provided in distinctive bottles (500 cc) placed in an individual macaque’s cage for 4 h. PTC solutions were prepared at concentrations of 1, 10, 100, 250, and 1000 μM in distilled water. PTC solution concentrations and positions were randomly changed to prevent position preferences. During the test, we monitored monkey behavior by video and checked for liquid spillage caused by accidents due to monkey interference. To prevent monkey interference, we covered the bottles, exposing only the feed-water nozzle as needed. The health of animals was carefully checked daily by the experimenter and keeper. This trial was conducted once a day every weekday and repeated 6 times for each concentration. A preference ratio was calculated as the ratio of PTC solution volume consumed to the total liquid volume consumed. Based on the mean preference ratio at each concentration, we fit the data to the equation: *f(x)* = 0.5/[1 + (*x*/EC_50_)^*h*^], where *x* was the PTC concentration, with the Levenberg–Marquardt algorithm using R v2.14.0.

### Analyses of *TAS2R38* coding and flanking regions in the Kii population

To elucidate the evolutionary background of *Mf-K* the non-taster *TAS2R38* allele, sequence variation in the *TAS2R38* gene (1,002 bp) and additional non-coding regions that flanked either side of this gene was examined. For this analysis, 40 DNA samples derived from the Kii population were used, including all individuals that had the *Mf-K* allele. We designed the primer sets shown in S4 Table to amplify the *TAS2R38* flanking region of Japanese macaques based on the genome sequence of rhesus macaques in 2 parts: the 5′-flanking region plus the *TAS2R38* coding region and the 3′-flanking region plus the *TAS2R38* coding region. The PCR extension time was 5 min and the other conditions were the same as those used for *TAS2R38* sequencing and haplotype inferences described above.

Haplotype names were assigned on the basis of the *TAS2R38* coding region. These flanking sequences were deposited in GenBank under the accession numbers AB907288–AB907300. Recombination in this flanking region was identified using GENECONV [51] and the recombination boundary in the 5′ region of *TAS2R38* was estimated. This region was excluded from analysis, and only a 6411 bp sequence (rheMac2, chr3: 179,402,166–179,408,638) that included the *TAS2R38* coding region was used for further analyses.

A median-joining network of evolutionary relationships among available haplotypes was constructed using NETWORK v4.6 [48]. Indel polymorphisms were weighted twice as much as substitution polymorphisms.

### Sequencing analysis of non-coding regions in the Kii population

To characterize the genetic diversity and migration rate of genes in the Kii population, the non-coding intergenic regions were analyzed in the Kii and 7 neighboring populations, Takahama, Jigokudani, Hagachi, Mie, Shiga, Arashiyama, and Minoo (Fig 1). Eight individuals from each population were investigated. Nine non-coding loci were randomly selected from the 27 regions described by Osada et al. [52], and they are listed in S4 Table along with the primers used for amplification and sequencing. The methods used for amplifying and sequencing a target region were the same as those described above. If a sample had more than 1 heterozygous indel in the target region, the sequences were not used for further analyses. Haplotypes were reconstructed from diploid sequences sets using PHASE v2.1 [42,43]. The non-coding sequences were deposited in GenBank under the accession numbers AB907231–AB907287. Summary statistics, including *π*, *θ*, *D*, and pairwise *F*_ST_, were determined using DnaSP v5.1 [44]. *Nm* values were calculated from the formula: *F*_ST_ = 1/(1 + 4*Nm*), where *N* and *m* represent the effective population size and migration rate, respectively [53].

## Supporting Information

S1 Fig Schematic of vector constructs used to analyze receptor activity. Red and gray squares indicate somatostatin (ssr3) and rhodopsin (bRh) tags, respectively. Blue lines indicate differences in amino acids compared with MfTAS2R38-A.
(PDF)

S2 Fig Gustatory responsiveness of each individual to various PTC solution concentrations. These Figs show the individual data summarized in Fig 4. Each point represents the mean ± standard error determined from 6 trials. The seven Japanese macaques have 3 genotypes at the TAS2R38 start codon: (A-D) ATG/ATG, (E-F) ACG/ACG and (G): ATG/ACG.
(PDF)

S3 Fig Segregating sites in 13 haplotypes of the *TAS2R38* flanking region. Segregating sites are divided into 3 parts: 5′-flanking, TAS2R38 coding, and 3′-flanking regions. Numbers above the segregating sites indicate the nucleotide position of each site and correspond to the last 5 positional digits in the rheMac2 genome (chr3;179,4xx,xxx). Dots indicate nucleotides identical to those in the top line sequence (A-1). Pound signs and asterisks indicate indel polymorphisms.
(PDF)

S1 Table Variable sites in 20 Japanese and 10 rhesus macaque *TAS2R38* alleles and their frequencies. (PDF)

S2 Table The number of *TAS2R38* alleles in 17 populations. (PDF)

S3 Table Variation in non-coding regions in 8 local populations of Japanese macaques. (PDF)

S4 Table PCR and sequencing primers used in this study. (PDF)
