# Supplementary material for: Rapid Expansion of Phenylthiocarbamide Non-Tasters among Japanese Macaques
Source: PLoS One. 2015 Jul 22;10(7):e0132016. doi: 10.1371/journal.pone.0132016 (PMC4511751; doi:10.1371/journal.pone.0132016)
Supplement: S1 Fig — Red and gray squares indicate somatostatin (ssr3) and rhodopsin (bRh) tags, respectively. Blue lines indicate differences in amino acids compared with MfTAS2R38-A. (PDF) [file pone.0132016.s001.pdf]

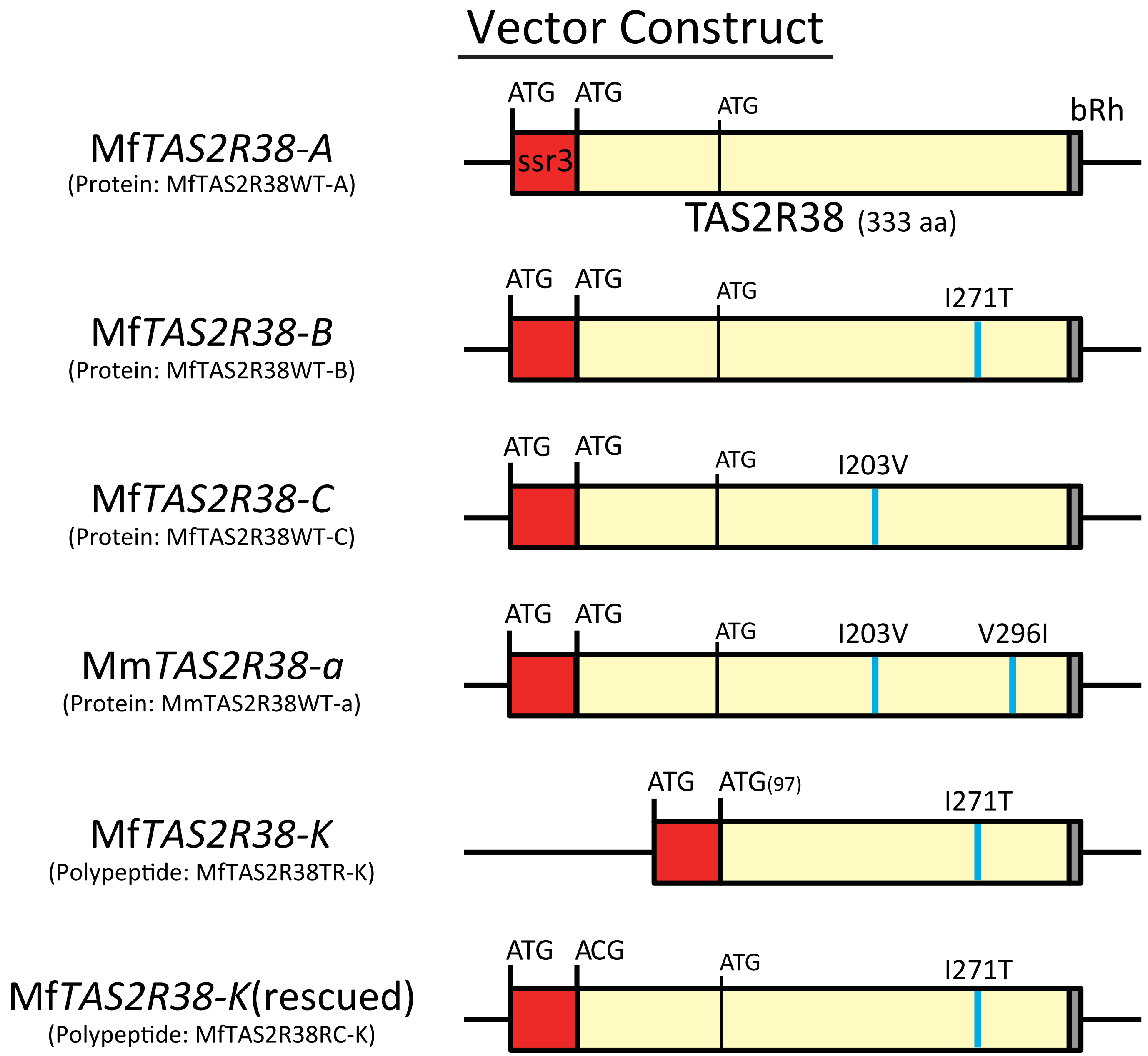


**S1 Fig. Schematic of vector constructs used to analyze receptor activity**. Red and gray squares indicate somatostatin (ssr3) and rhodopsin (bRh) tags, respectively. Blue lines indicate differences in amino acids compared with MfTAS2R38-A.
