# Supplementary material for: Rapid Expansion of Phenylthiocarbamide Non-Tasters among Japanese Macaques
Source: PLoS One. 2015 Jul 22;10(7):e0132016. doi: 10.1371/journal.pone.0132016 (PMC4511751; doi:10.1371/journal.pone.0132016)
Supplement: S2 Fig — These Figs show the individual data summarized in Fig 4. Each point represents the mean ± standard error determined from 6 trials. The seven Japanese macaques have 3 genotypes at the TAS2R38 start codon: (A-D) ATG/ATG, (E-F) ACG/ACG and (G): ATG/ACG. (PDF) [file pone.0132016.s002.pdf]

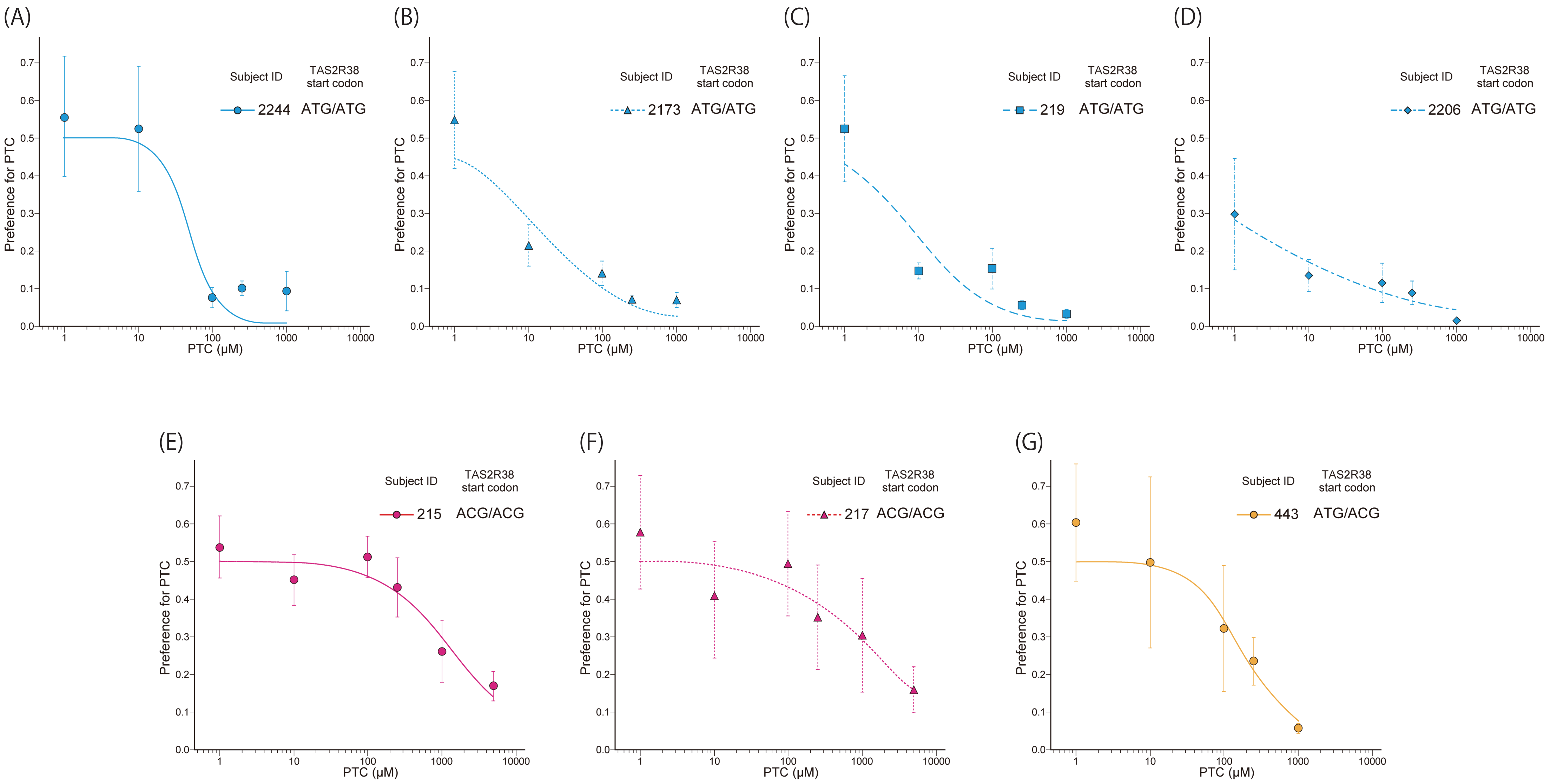

S2 Fig. Gustatory responsiveness of each individual to various PTC solution concentrations. These figures show the individual data summarized in Fig 4. Each point represents the mean ± standard error determined from 6 trials. The seven Japanese macaques have 3 genotypes at the TAS2R38 start codon: (A-D) ATG/ATG, (E-F) ACG/ACG and (G): ATG/ACG.
