# Supplementary material for: Rapid Expansion of Phenylthiocarbamide Non-Tasters among Japanese Macaques
Source: PLoS One. 2015 Jul 22;10(7):e0132016. doi: 10.1371/journal.pone.0132016 (PMC4511751; doi:10.1371/journal.pone.0132016)
Supplement: S1 Table — (PDF) [file pone.0132016.s004.pdf]

S1 Table. Variable sites in 20 Japanese and 10 rhesus macaque *TAS2R38* alleles and their frequencies.

Nucleotide Change		T2C	T9G	C10G	G19A	G164T,C	G191A	C243T	T349C	G368A	G607A	G704A	C736T	T812C	G886A	C930G	A959C	C969T	C972T	C992T		
Amino Acid Change		M1T	T3T	L4V	V7I	C55F,S	R64Q	F81F	Y117H	R123H	V203I	R235H	L246F	I271T	V296I	T310T	K320T	A323A	D324D	T331I		
	Paha	T	T	C	A	G	G	C	T	A	G	G	C	T	G	C	A	C	C	C	*N*	Freq.(%)
Japanese macaques	*Mf-A*	T	T	C	G	G	G	C	T	G	A	G	C	T	G	C	A	C	C	C	519	43.47
	Mf-B	.	.	.	.	.	.	.	.	.	.	.	.	C	.	.	.	.	.	.	373	31.24
	Mf-C	.	.	.	.	.	.	.	.	.	G	.	.	.	A	.	.	.	.	.	77	6.45
	Mf-D	.	.	G	.	.	.	.	.	.	.	.	.	.	.	.	.	.	.	.	19	1.59
	Mf-E	.	.	G	.	.	.	.	.	.	G	.	.	.	A	.	.	.	.	.	25	2.09
	Mf-F	.	.	G	A	.	.	.	.	.	G	.	.	.	A	.	.	.	.	.	29	2.43
	Mf-G	.	.	.	.	.	.	T	.	.	G	.	.	.	A	.	.	.	.	.	10	0.84
	Mf-H	.	.	G	.	.	.	.	C	.	G	A	.	.	A	.	.	.	.	.	15	1.26
	Mf-I	.	.	.	.	.	.	.	.	.	.	.	.	.	.	.	.	T	.	.	21	1.76
	Mf-J	.	.	.	.	.	.	.	.	A	.	.	.	.	.	.	.	.	.	.	8	0.67
	Mf-K	C	.	.	.	.	.	.	.	.	.	.	.	C	.	.	.	.	.	.	23	1.93
	Mf-L	.	.	G	A	.	.	.	.	.	.	.	.	.	.	.	.	.	.	.	17	1.42
	Mf-M	.	.	.	.	T	.	.	.	.	.	.	.	.	.	.	.	.	.	.	10	0.84
	Mf-N	.	G	.	.	.	.	.	.	.	.	.	.	C	.	.	.	.	.	.	4	0.34
	Mf-O	.	.	.	.	.	.	.	.	.	.	.	.	C	.	.	C	.	.	.	33	2.76
	Mf-P	.	.	G	A	.	.	.	.	.	.	.	.	C	.	.	.	.	.	.	2	0.17
	Mf-Q	.	.	.	.	.	A	.	.	.	.	.	.	.	.	.	.	.	.	.	3	0.25
	Mf-R	.	G	.	.	.	.	.	.	.	G	.	.	.	A	.	.	.	.	.	4	0.34
	Mf-S	.	.	.	A	.	.	.	.	.	G	.	.	.	A	.	.	.	.	.	1	0.08
	Mf-T	.	.	G	.	.	.	.	C	.	G	.	.	.	A	.	.	.	.	.	1	0.08
rhesus macaques[a]	*Mm-a*	.	.	.	.	.	.	.	.	.	G	.	.	.	.	.	.	.	.	.	13	12.04
	Mm-b	.	.	.	.	.	.	.	.	.	G	.	.	.	.	.	.	.	T	T	39	36.11
	Mm-d	.	.	.	A	.	.	.	.	.	G	.	.	.	.	.	.	.	.	.	11	10.19
	Mm-f	.	.	.	.	.	.	.	.	.	G	.	T	.	.	.	.	.	T	T	4	3.70
	Mm-g	.	.	.	A	C	.	.	.	.	G	.	.	.	.	.	.	.	.	.	9	8.33
	Mm-i	.	.	.	A	.	.	.	.	.	.	.	.	.	.	.	.	.	T	T	18	16.67
	Mm-j	.	.	.	A	C	.	.	.	.	G	.	.	.	.	G	.	.	.	.	6	5.56
	Mm-k	.	.	.	A	.	.	.	.	.	G	.	.	.	.	G	.	.	.	.	4	3.70
	Mm-l	.	.	.	.	.	.	.	.	.	G	.	.	.	.	G	.	.	.	.	3	2.78
	Mm-m	.	.	.	.	.	.	.	.	.	G	.	.	.	.	G	.	.	.	.	1	0.93

[a]Three rhesus macaque alleles, Mm-c, -e, and -h, defined in our previous study (Suzuki et al. 2010) were excluded, as the haplotype phase was inferred by other methods.
