# Supplementary material for: Rapid Expansion of Phenylthiocarbamide Non-Tasters among Japanese Macaques
Source: PLoS One. 2015 Jul 22;10(7):e0132016. doi: 10.1371/journal.pone.0132016 (PMC4511751; doi:10.1371/journal.pone.0132016)
Supplement: S2 Table — (PDF) [file pone.0132016.s005.pdf]

S2 Table. The number of *TAS2R38* alleles in 17 populations.

	Allele group[a]	SMK	KKZ	NMT	THM	JGD	HGC	OKZ	MIE	SHG	ARY	MNO	KII	WKS	SHD	KAM	TKO	KOS	
Mf-A	(1)	84	0	3	26	24	15	2	84	31	42	62	32	21	2	3	0	88	519
Mf-B	(1)	49	18	32	10	47	14	4	23	16	13	3	17	25	20	11	50	21	373
Mf-C	(3)	0	0	0	6	1	1	0	14	8	0	3	0	1	0	1	0	42	77
Mf-D	(3)	1	0	0	1	0	0	0	0	5	0	0	0	12	0	0	0	0	19
Mf-E	(3)	0	0	0	7	0	0	0	7	7	3	0	0	0	0	1	0	0	25
Mf-F	(3)	0	0	0	4	0	0	0	6	2	0	0	1	11	0	5	0	0	29
Mf-G	(2)	0	0	0	0	0	0	0	0	0	0	0	0	10	0	0	0	0	10
Mf-H	(3)	0	0	0	0	0	0	0	9	0	0	4	0	2	0	0	0	0	15
Mf-I	(3)	0	0	0	0	8	0	2	0	0	0	0	0	0	0	0	0	11	21
Mf-J	(3)	0	0	0	0	0	0	0	2	0	0	0	6	0	0	0	0	0	8
Mf-K	(2)	0	0	0	0	0	0	0	0	0	0	0	23	0	0	0	0	0	23
Mf-L	(3)	0	0	0	0	0	0	0	8	1	0	0	0	0	0	0	8	0	17
Mf-M	(2)	0	0	0	0	0	0	0	0	0	0	10	0	0	0	0	0	0	10
Mf-N	(2)	0	0	4	0	0	0	0	0	0	0	0	0	0	0	0	0	0	4
Mf-O	(3)	32	0	1	0	0	0	0	0	0	0	0	0	0	0	0	0	0	33
Mf-P	(3)	0	0	0	0	0	0	0	1	0	0	0	0	0	0	1	0	0	2
Mf-Q	(3)	0	0	0	0	0	0	0	2	0	0	0	1	0	0	0	0	0	3
Mf-R	(2)	0	0	0	0	0	0	0	0	4	0	0	0	0	0	0	0	0	4
Mf-S	(2)	0	0	0	0	0	0	0	0	0	0	0	0	0	0	1	0	0	1
Mf-T	(2)	0	0	0	0	0	0	0	0	0	0	0	0	0	0	1	0	0	1
		166	18	40	54	80	30	8	156	74	58	82	80	82	22	24	58	162	1194

[a]Allele groups are: (1) major alleles that observed in many populations, (2) population-specific alleles, and (3) alleles shared by several populations.
