# Supplementary material for: Rapid Expansion of Phenylthiocarbamide Non-Tasters among Japanese Macaques
Source: PLoS One. 2015 Jul 22;10(7):e0132016. doi: 10.1371/journal.pone.0132016 (PMC4511751; doi:10.1371/journal.pone.0132016)
Supplement: S3 Table — (PDF) [file pone.0132016.s006.pdf]

S3 Table. Variations in non-coding regions in 8 local populations of Japanese macaques.

						Takahama						Jigokudani						Hagachi						Mie					
	Locus	chr	Length	S[a]	h[b]	n[c]	S[a]	h[b]	π[d]	θ[e]	D[f]	n[c]	S[a]	h[b]	π[d]	θ[e]	D[f]	n[c]	S[a]	h[b]	π[d]	θ[e]	D[f]	n[c]	S[a]	h[b]	π[d]	θ[e]	D[f]
1	*IGS03*	2	764	5	6	8	0	1	0.000	0.000	NA	8	0	1	0.000	0.000	NA	8	2	3	0.059	0.079	-0.65	8	1	2	0.031	0.039	-0.45
2	*IGS05*	3	666	4	5	8	1	2	0.035	0.045	-0.45	8	2	3	0.079	0.091	-0.33	8	1	2	0.019	0.045	-1.16	8	0	1	0.000	0.000	NA
3	*IGS09*	4	760	7	7	8	1	2	0.060	0.040	1.03	8	4	3	0.260	0.159	2.00*	8	5	4	0.125	0.198	-1.22	8	5	5	0.234	0.198	0.59
4	*IGS13*	7	795	12	13	8	2	3	0.057	0.076	-0.65	8	3	3	0.131	0.114	0.44	8	4	4	0.104	0.152	-0.99	8	3	4	0.061	0.114	-1.35
5	*IGS15*	8	707	5	6	5	0	1	0.000	0.000	NA	5	3	4	0.107	0.150	-1.03	8	2	3	0.064	0.085	-0.65	5	0	1	0.000	0.000	NA
6	*IGS19*	10	653	4	5	8	0	1	0.000	0.000	NA	8	2	3	0.080	0.092	-0.33	8	2	3	0.117	0.092	0.70	8	1	2	0.019	0.046	-1.16
7	*IGS21*	11	753	2	3	8	1	2	0.061	0.040	1.03	8	0	1	0.000	0.000	NA	8	1	2	0.017	0.040	-1.16	8	0	1	0.000	0.000	NA
8	*IGS25*	16	716	2	3	8	1	2	0.017	0.042	-1.16	8	1	2	0.064	0.042	1.03	8	1	2	0.064	0.042	1.03	8	1	2	0.017	0.042	-1.16
9	*IGS27*	20	715	12	9	8	5	3	0.298	0.211	1.37	8	6	4	0.221	0.253	-0.43	8	8	5	0.338	0.337	0.01	8	7	5	0.372	0.295	0.92
	average		725.4	5.9	6.3		1.2	1.9	0.059	0.050	-		2.3	2.7	0.105	0.100	-		2.9	3.1	0.101	0.119	-		2.0	2.6	0.082	0.082	-

						Shiga						Arashiyama						Minoo						Kii						average			
	Locus	chr	Length	S[a]	h[b]	n[c]	S[a]	h[b]	π[d]	θ[e]	D[f]	n[c]	S[a]	h[b]	π[d]	θ[e]	D[f]	n[c]	S[a]	h[b]	π[d]	θ[e]	D[f]	n[c]	S[a]	h[b]	π[d]	θ[e]	D[f]	S[a]	h[b]	π[d]	θ[e]
1	*IGS03*	2	764	5	6	6	2	3	0.061	0.087	-0.85	8	0	1	0.000	0.000	NA	8	0	1	0.000	0.000	NA	8	1	2	0.043	0.039	0.16	0.7	1.8	0.024	0.031
2	*IGS05*	3	666	4	5	8	1	2	0.019	0.045	-1.16	8	1	2	0.019	0.045	-1.16	8	0	1	0.000	0.000	NA	8	2	3	0.038	0.091	-1.50	1.1	2.0	0.026	0.045
3	*IGS09*	4	760	7	7	8	4	3	0.224	0.159	1.28	8	3	2	0.181	0.119	1.51	8	3	2	0.158	0.119	0.95	8	3	3	0.209	0.119	2.20*	3.3	3.0	0.181	0.139
4	*IGS13*	7	795	12	13	8	4	5	0.102	0.152	-1.03	8	2	3	0.080	0.076	0.13	8	3	4	0.108	0.114	-0.15	8	2	2	0.059	0.076	-0.58	2.9	3.5	0.088	0.109
5	*IGS15*	8	707	5	6	5	1	2	0.028	0.050	-1.11	5	1	2	0.028	0.050	-1.11	5	1	2	0.050	0.050	0.01	4	0	1	0.000	0.000	NA	1.1	2.0	0.035	0.048
6	*IGS19*	10	653	4	5	8	2	3	0.038	0.092	-1.50	8	2	3	0.038	0.092	-1.50	8	1	2	0.070	0.046	1.03	8	1	2	0.019	0.046	-1.16	1.4	2.4	0.048	0.063
7	*IGS21*	11	753	2	3	8	1	2	0.061	0.040	1.03	8	1	2	0.053	0.040	0.65	8	1	2	0.070	0.040	1.47	8	1	2	0.043	0.040	0.16	0.9	1.8	0.038	0.030
8	*IGS25*	16	716	2	3	8	1	2	0.017	0.042	-1.16	8	0	1	0.000	0.000	NA	8	0	1	0.000	0.000	NA	8	0	1	0.000	0.000	NA	0.6	1.6	0.022	0.026
9	*IGS27*	20	715	12	9	8	8	4	0.357	0.337	0.21	8	4	2	0.256	0.169	1.63	8	7	5	0.339	0.295	0.53	8	5	3	0.274	0.211	0.99	6.1	3.9	0.307	0.264
	average		725.4	5.9	6.3		2.7	2.9	0.101	0.112	-		1.6	2.0	0.073	0.066	-		1.8	2.2	0.088	0.074	-		1.7	2.1	0.076	0.069	-	2.0	2.4	0.085	0.084

[a]Number of polymorphic sites.

[b]Number of haplotypes.

[c]Number of individuals.

[d]Nucleotide diversity per site (%).

[e]Watterson's θ per site (%).

[f]Tajima's D. Two-sided Tajima's D test was performed using coalescent simulations under 10,000 replicates, assuming no recombination and a Poisson distribution of mutations along lineages. * indicates P < 0.05.
