# Supplementary material for: Rapid Expansion of Phenylthiocarbamide Non-Tasters among Japanese Macaques
Source: PLoS One. 2015 Jul 22;10(7):e0132016. doi: 10.1371/journal.pone.0132016 (PMC4511751; doi:10.1371/journal.pone.0132016)
Supplement: S4 Table — (PDF) [file pone.0132016.s007.pdf]

S4 Table. PCR and sequencing primers used in this study.

Locus		Position in rheMac2 genome	Forward Primer		Reverse Primer		
			Name	Sequence	Name	Sequence	Annealing Temperature
TAS2R38	PCR	chr3:179,407,166-179,408,167	Mm-TAS2R38-F	5'-GCCAACTAGAGAAGAGAAGTAGA-3'	Mm-TAS2R38-R	5'-GACTCACAGGCGTATTAAGG-3'	56 ºC
	Sequencing		Mm-TAS2R38_inner-F	5'-CAGCCTGCTCTACTGCTCCAA-3'	Mm-TAS2R38_inner-R	5'-AGTCTGCCAAAAAAGCACCAAA-3'	-
TAS2R38_5'-flanking	PCR	chr3:179,407,123-179,413,514	TAS2R38_5'-flanking-F	5'-TGTCTCGCTGTTCTAGGAAT-3'	Mm-TAS2R38-R	5'-GACTCACAGGCGTATTAAGG-3'	60 ºC
	Sequencing		chr3-179408908-F	5'-CTTCATGACCCAGTGCTAGT-3'	chr3-179408140-R	5'-CAGTGCAGACGTGAGTTAGA-3'	-
	Sequencing		chr3-179409668-F	5'-TTGGAGTCTCTGTGCCTACT-3'	chr3-179408769-R	5'-GTTCCTTTCCATGTGTCAGT-3'	-
	Sequencing		chr3-179410245-F	5'-GCAAGGGATACGATTCATAG-3'	chr3-179409440-R	5'-GTTTCCCAATTCCCTTACTC-3'	-
	Sequencing		chr3-179410964-F	5'-TAGCCTGAATTTTTCCAGAG-3'	chr3-179410226-R	5'-CTATGAATCGTATCCCTTGC-3'	-
	Sequencing		chr3-179411606-F	5'-GACTGGGTGAGGAATATCAA-3'	chr3-179410873-R	5'-TGGCCTTCCAATATTTCTAA-3'	-
	Sequencing		chr3-179412332-F	5'-TCTGACTCCCAGGTTCATAC-3'	chr3-179411582-R	5'-TGCTCTTGATATTCCTCACC-3'	-
	Sequencing		chr3-179412873-F	5'-CTGTCAAGCTAACGAAGACC-3'	chr3-179412183-R	5'-TTCAGTAATCGAGACCATCC-3'	-
TAS2R38_3'-flanking	PCR	chr3:179,402,038-179,408,214	Mm-TAS2R38-F	5'-GCCAACTAGAGAAGAGAAGTAGA-3'	TAS2R38_5'-flanking-R	5'-CAAGAAATCTGCCCTCATAG-3'	61 ºC
	Sequencing		chr3-179407176-F	5'-CACTGTGCTGAGAATGGATA-3'	chr3-179406340-R	5'-GAGAATGCCAAATCTCTACAA-3'	-
	Sequencing		chr3-179406474-F	5'-GTGTGGACTTTCTGGCTATG-3'	chr3-179405808-R	5'-GGGCATCTAGAAGAGTGGAT-3'	-
	Sequencing		chr3-179405797-F	5'-GAGATGGAGCATCTTTTCAT-3'	chr3-179405141-R	'-ACAACTTAATGTAAAACAGTTGTG-[illegible]	-
	Sequencing		chr3-179404909-F	5'-TACAATGCTTGTGCTTTTTG-3'	chr3-179405010-R	5'-CAATGCATATATCAGACAAAGG-3'	-
	Sequencing		chr3-179404567-F	5'-TTTGGATTATGGTAGATTACTGG-3'	chr3-179404589-R	5'-TGGACAAATAGATCACTGGA-3'	-
	Sequencing		chr3-179404018-F	5'-TCAGATGTAAAAAGCCCATT-3'	chr3-179404189-R	5'-GGGCATATGTATGGAGAAAG-3'	-
	Sequencing		chr3-179403523-F	5'-TTCATGAAGGTTTGCTTTTT-3'	chr3-179403269-R	5'-TTGATGAATCACTGGCTAGA-3'	-
	Sequencing		chr3-179403082-F	5'-CTTTACGTTTCCTGCTCAAC-3'	chr3-179402324-R	5'-TACAGCAATGCAAGAATGAC-3'	-
IGS03	PCR and Sequencing	chr2:5,175,371-5,176,134	Mm-IGS03-F	5'-TGCTCTACCTGTGCGAATTG-3'	Mm-IGS03-R	5'-ATGAATTGTTCACCCCCAAA-3'	60 ºC
IGS05		chr3:14,376,060-14,376,727	Mm-IGS05-F	5'-TGGCAGCCAGTCTTCTCTTT-3'	Mm-IGS05-R	5'-CACCTAGGGCCACACTGAAT-3'	60 ºC
IGS09		chr4:141,712,948-141,713,707	Mm-IGS09-F	5'-TCCTTCTTCCAACAGACCAGA-3'	Mm-IGS09-R	5'-CCACCAGGCTCCTCTCATTA-3'	60 ºC
IGS13		chr7:101,026,368-101,027,162	Mm-IGS13-F	5'-CCGGTGCAGCTAATGTCTTT-3'	Mm-IGS13-R	5'-TGGCCATGTAATGAGTTCCA-3'	60 ºC
IGS15		chr8:98,384,350-98,385,056	Mm-IGS15-F	5'-TGGTTTTGACAGGTGACTGC-3'	Mm-IGS15-R	5'-ATGGTGACTCACTGCTTGGA-3'	60 ºC
IGS19		chr10:71,411,234-71,411,886	Mm-IGS19-F	5'-AGATGTGAGCCTGAGCCTGT-3'	Mm-IGS19-R	5'-GCTGAAGCAGAACCCAGAAC-3'	60 ºC
IGS21		chr11:99,301,129-99,301,881	Mm-IGS21-F	5'-GGGAACTGTCCTGATTGCAT-3'	Mm-IGS21-R	5'-CCCCTTCATCTTCCTCCTTC-3'	60 ºC
IGS25		chr16:26,856,824-26,857,539	Mm-IGS25-F	5'-GCCATAAAGCACACTGCTCA-3'	Mm-IGS25-R	5'-CATTCAGCTTTTGCAGTGGA-3'	60 ºC
IGS27		chr20:17,422,254-17,422,968	Mm-IGS27-F	5'-GGGGTAGAGGGAACATGGAT-3'	Mm-IGS27-R	5'-GGGGTTCTTTTGGGGTATGT-3'	60 ºC
