# Supplementary material for: Rapid Expansion of Phenylthiocarbamide Non-Tasters among Japanese Macaques
Source: PLoS One. 2015 Jul 22;10(7):e0132016. doi: 10.1371/journal.pone.0132016 (PMC4511751; doi:10.1371/journal.pone.0132016)
Supplement: S3 Fig — Segregating sites are divided into 3 parts: 5′-flanking, TAS2R38 coding, and 3′-flanking regions. Numbers above the segregating sites indicate the nucleotide position of each site and correspond to the last 5 positional digits in the rheMac2 genome (chr3;179,4xx,xxx). Dots indicate nucleotides identical to those in the top line sequence (A-1). Pound signs and asterisks indicate indel polymorphisms. (PDF) [file pone.0132016.s003.pdf]

Coding allele	Flanking haplotyp	*N*	5' flanking	5' flanking	*TAS2R38*	3' flanking
			111111111111111111111111111111111111111111000000000000	000000	00000000	00000000000000000
			222222222222222111111111110000000000000000999999999888	888888	88877777	66555554444432222
			333333333322210997776632009987776655421100888432100966	443222	11198532	80533006541079732
			988765554398502935213235649446559042767543985126152643	831987	65470658	09620656759294568
			241664324710907418227979692743549895724197984029816039	368048	68970162	53304338990266627
Mf-A	*A-1*	15	AACTTACACCCCCCAAC#CGGTCACTCGACCATGATTTAGTCAC*ATTAGG#GC	TCGGTA	TCGGGATG	AATGTT#C#TCTTCATA
Mf-A	*A-2*	8	GG.AGCGG...GT..CT#..A.T.TG.A..TGC.GCA...AGG.#C.GGT.#A.	C..CGG	....	......#.#......CG
Mf-B	*B-1*	13	GG.GGCGGT..G...CT#..A.T.TG.A...GC.GCA...AGG.#C..GTA*..	C..CGG	......C.	...T..#T#.....T..
Mf-J	*J-1*	6	GG.GGCGG...G...CT#..A.T.TG.A...GC.GCA...AGG.#C..GTA*..	C..CGG	....A...	...T.A#.#........
Mf-Q	*Q-1*	1	GG.GGCGG...G...CT#..A.T.TG.A...GC.GCA...AGGT#C..GTA*..	C..CGG	...A....	.C.TC.#.#.G.C....
Mf-A	*A-3*	4	..T..........TG.T*ATA..GTG.ACT.GCAGC.CCA..G.#.A.GT.#..	C..CGG	....	G..T.A#.#C.......
Mf-A	*A-4*	2	..T..........TG.T*ATA...TG.ACT.GCAGC.C.A..G.#.A.GT.#AT	C..CGG	....	..AT..*.*..A.T.C.
Mf-A	*A-5*	1	..T..........TG.T*ATA...TG.ACT.GCA.C.C.A..G.#.A.GT.#AT	C..CGG	....	...T..#.#........
Mf-A	*A-6*	1	..T.......T..TG.T*ATAC..TG.ACT.GCAGC.C.A..G.#.A.GT.#A.	C..CGG	....	...T..#.#........
Mf-A	*A-7*	1	..T..........TG.T*ATA...TG.ACT.GCA.C.C.A..G.#.A.GT.#..	C..CGG	....	...T..*.#........
Mf-B	*B-2*	4	..T.....GT...TG.T*ATA...TG.ACT.GCAGC.C.A..G.#.A.GT.#A.	C..CGG	......C.	.C.TC.#.#.G......
Mf-F	*F-1*	1	..T.......T..TG.T*ATAC..TGTACT.GCAGC.C.A..G.#.A.GT.#A.	C..CGG	.GA..G.A	...T.A#.#........
Mf-K	*K-1*	23	..T..........TG.T*ATA..GTG.ACT.GCAGC.C.A..G.#.A.GT.#A.	CTCCGG	C.....C.	..AT..*.*........
			Excluded region for haplotype network	Analyzed region for haplotype network		

S3 Fig. **Segregating sites in 13 haplotypes of the *TAS2R38* flanking region**. Segregating sites are divided into 3 parts: 5′-flanking, TAS2R38 coding, and 3′-flanking regions. Numbers above the segregating sites indicate the nucleotide position of each site and correspond to the last 5 positional digits in the rheMac2 genome (chr3;179,4xx,xxx). Dots indicate nucleotides identical to those in the top line sequence (A-1). Pound signs and asterisks indicate indel polymorphisms.
